# THE CONCISE GUIDE TO PHARMACOLOGY 2017/18: Voltage‐gated ion channels

**DOI:** 10.1111/bph.13884

**Published:** 2017-10-21

**Authors:** Stephen PH Alexander, Jörg Striessnig, Eamonn Kelly, Neil V Marrion, John A Peters, Elena Faccenda, Simon D Harding, Adam J Pawson, Joanna L Sharman, Christopher Southan, Jamie A Davies, CGTP Collaborators

**Affiliations:** ^1^ School of Life Sciences University of Nottingham Medical School Nottingham NG7 2UH UK; ^2^ Pharmacology and Toxicology Institute of Pharmacy, University of Innsbruck A‐6020 Innsbruck Austria; ^3^ School of Physiology, Pharmacology and Neuroscience University of Bristol Bristol BS8 1TD UK; ^4^ Neuroscience Division, Medical Education Institute, Ninewells Hospital and Medical School University of Dundee Dundee DD1 9SY UK; ^5^ Centre for Integrative Physiology University of Edinburgh Edinburgh EH8 9XD UK

## Abstract

The Concise Guide to PHARMACOLOGY 2017/18 provides concise overviews of the key properties of nearly 1800 human drug targets with an emphasis on selective pharmacology (where available), plus links to an open access knowledgebase of drug targets and their ligands (www.guidetopharmacology.org), which provides more detailed views of target and ligand properties. Although the Concise Guide represents approximately 400 pages, the material presented is substantially reduced compared to information and links presented on the website. It provides a permanent, citable, point‐in‐time record that will survive database updates. The full contents of this section can be found at http://onlinelibrary.wiley.com/doi/10.1111/bph.13884/full. Voltage‐gated ion channels are one of the eight major pharmacological targets into which the Guide is divided, with the others being: G protein‐coupled receptors, ligand‐gated ion channels, other ion channels, nuclear hormone receptors, catalytic receptors, enzymes and transporters. These are presented with nomenclature guidance and summary information on the best available pharmacological tools, alongside key references and suggestions for further reading. The landscape format of the Concise Guide is designed to facilitate comparison of related targets from material contemporary to mid‐2017, and supersedes data presented in the 2015/16 and 2013/14 Concise Guides and previous Guides to Receptors and Channels. It is produced in close conjunction with the Nomenclature Committee of the Union of Basic and Clinical Pharmacology (NC‐IUPHAR), therefore, providing official IUPHAR classification and nomenclature for human drug targets, where appropriate.

## Conflict of interest

The authors state that there are no conflicts of interest to declare.

## Family structure

S161 CatSper and Two‐Pore channels

S162 Cyclic nucleotide‐regulated channels

S164 Potassium channels

S165 Calcium‐ and sodium‐activated potassium channels

S166 Inwardly rectifying potassium channels

S169 Two P domain potassium channels

S171 Voltage‐gated potassium channels

S175 Ryanodine receptors

S176 Transient Receptor Potential channels

S186 Voltage‐gated calcium channels

S188 Voltage‐gated proton channel

S189 Voltage‐gated sodium channels

## CatSper and Two‐Pore channels

### Overview

CatSper channels (CatSper1‐4, **nomenclature as agreed by** **NC‐IUPHAR** **[** **69** **]**) are putative 6TM, voltage‐gated, calcium permeant channels that are presumed to assemble as a tetramer of *α*‐like subunits and mediate the current I_CatSper_[193]. In mammals, CatSper subunits are structurally most closely related to individual domains of voltage‐activated calcium channels (Ca_v_) [349]. CatSper1 [349], CatSper2 [341] and CatSpers 3 and 4 [173, 245, 338], in common with a putative 2TM auxiliary CatSper*β* protein [242] and two putative 1TM associated CatSper*γ* and CatSper*δ* proteins [64, 434], are restricted to the testis and localised to the principle piece of sperm tail.

Two‐pore channels (TPCs) are structurally related to CatSpers, Ca_V_s and Na_V_s. TPCs have a 2x6TM structure with twice the number of TMs of CatSpers and half that of Ca_V_s. There are three animal TPCs (TPC1‐TPC3). Humans have TPC1 and TPC2, but not TPC3. TPC1 and TPC2 are localized in endosomes and lysosomes [43]. TPC3 is also found on the plasma membrane and forms a voltage‐activated, non‐inactivating Na^+^ channel [44]. All the three TPCs are Na^+^‐selective under whole‐cell or whole‐organelle patch clamp recording [45, 46, 457]. The channels may also conduct Ca^2+^[272].

**Table:** 

Nomenclature	CatSper1	CatSper2	CatSper3	CatSper4
HGNC, UniProt	CATSPER1, Q8NEC5	CATSPER2, Q96P56	CATSPER3, Q86XQ3	CATSPER4, Q7RTX7
Activators	CatSper1 is constitutively active, weakly facilitated by membrane depolarisation, strongly augmented by intracellular alkalinisation. In human, but not mouse, spermatozoa progesterone (EC_50_ ∼8 nM) also potentiates the CatSper current (I_CatSper_) [239, 390]	–	–	–
Channel blockers	ruthenium red (pIC_50_ 5) [193] – Mouse, HC‐056456 (pIC_50_ 4.7) [50], Cd^2+^ (pIC_50_ 3.7) [193] – Mouse, Ni^2+^ (pIC_50_ 3.5) [193] – Mouse	–	–	–
Selective channel blockers	NNC55‐0396 (pIC_50_ 5.7) [‐80mV – 80mV] [239, 390], mibefradil (pIC_50_ 4.4–4.5) [390]	–	–	–
Functional Characteristics	Calcium selective ion channel (Ba^2+^>Ca^2+^≫Mg^2+^≫Na^+^); quasilinear monovalent cation current in the absence of extracellular divalent cations; alkalinization shifts the voltage‐dependence of activation towards negative potentials [V_½_ @ pH 6.0 = +87 mV (mouse); V_½_ @ pH 7.5 = +11mV (mouse) or pH 7.4 = +85 mV (human)]; required for I_CatSper_ and male fertility (mouse and human)	Required for I_CatSper_ and male fertility (mouse and human)	Required for I_CatSper_ and male fertility (mouse)	Required for I_CatSper_ and male fertility (mouse)

**Table:** 

Nomenclature	TPC1	TPC2
HGNC, UniProt	TPCN1, Q9ULQ1	TPCN2, Q8NHX9
Activators	phosphatidyl (3,5) inositol bisphosphate (pEC_50_ 6.5) [45]	phosphatidyl (3,5) inositol bisphosphate (pEC_50_ 6.4) [439]
Channel blockers	verapamil (pIC_50_ 4.6) [45], Cd^2+^ (pIC_50_ 3.7) [45]	verapamil (pIC_50_ 5) [439]
Functional Characteristics	Organelle voltage‐gated Na^+^‐selective channel (Na^+^≫K^+^≫Ca^2+^); Required for the generation of action potential‐like long depolarization in lysosomes. Voltage‐dependence of activation is sensitive to luminal pH (determined from lysosomal recordings). *ψ*_1/2_ @ pH4.6 = +91 mV; *ψ*_1/2_ @ pH6.5 = +2.6 mV. Maximum activity requires PI(3,5)P2 and reduced [ATP]	Organelle voltage‐independent Na^+^‐selective channel (Na^+^≫K^+^≫Ca^2+^). Sensitive to the levels of PI(3,5)P2. Activated by decreases in [ATP] or depletion of extracellular amino acids

### Comments

CatSper channel subunits expressed singly, or in combination, fail to functionally express in heterologous expression systems [341, 349]. The properties of CatSper1 tabulated above are derived from whole cell voltage‐clamp recordings comparing currents endogenous to spermatozoa isolated from the corpus epididymis of wild‐type andCatsper1^(‐/‐)^ mice [193] and also mature human sperm [239, 390]. I_CatSper_ is also undetectable in the spermatozoa of Catsper2^(‐/‐)^,Catsper3^(‐/‐)^, Catsper4^(‐/‐)^, or CatSper*δ* ^(‐/‐)^ mice, and CatSper 1 associates with CatSper 2, 3, 4, *β*, *γ*, and *δ*[64, 242, 338]. Moreover, targeted disruption of Catsper1, 2, 3, 4, or *δ* genes results in an identical phenotype in which spermatozoa fail to exhibit the hyperactive movement (whip‐like flagellar beats) necessary for penetration of the egg cumulus and zona pellucida and subsequent fertilization. Such disruptions are associated with a deficit in alkalinization and depolarization‐evoked Ca^2+^ entry into spermatozoa [51, 64, 338]. Thus, it is likely that the CatSper pore is formed by a heterotetramer of CatSpers1‐4 [338] in association with the auxiliary subunits (*β*, *γ*, *δ*) that are also essential for function [64]. CatSper channels are required for the increase in intracellular Ca^2+^ concentration in sperm evoked by egg zona pellucida glycoproteins [457]. Mouse and human sperm swim against the fluid flow and Ca^2+^ signaling through CatSper is required for the rheotaxis [268]. In vivo, CatSper1‐null spermatozoa cannot ascend the female reproductive tracts efficiently [65, 151]. It has been shown that CatSper channels form four linear Ca^2+^ signaling domains along the flagella, which orchestrate capacitation‐associated tyrosine phosphorylation [65].The driving force for Ca^2+^ entry is principally determined by a mildly outwardly rectifying K^+^ channel (KSper) that, like CatSpers, is activated by intracellular alkalinization [283]. Mouse KSper is encoded by mSlo3, a protein detected only in testis [262, 283, 478]. In human sperm, such alkalinization may result from the activation of H_v_1, a proton channel [240]. Mutations in CatSpers are associated with syndromic and non‐syndromic male infertility [144]. In human ejaculated spermatozoa, progesterone (<50 nM) potentiates the CatSper current by a non‐genomic mechanism and acts synergistically with intracellular alkalinisation [239, 390]. Sperm cells from infertile patients with a deletion in CatSper2 gene lack I_CatSper_ and the progesterone response [375]. In addition, certain prostaglandins (e.g. PGF_1*α*_, PGE_1_) also potentiate CatSper mediated currents [239, 390].

In human sperm, CatSper channels are also activated by various small molecules including endocrine disrupting chemicals (EDC) and proposed as a polymodal sensor [39, 39].

TPCs are the major Na^+^ conductance in lysosomes; knocking out TPC1 and TPC2 eliminates the Na^+^ conductance and renders the organelle's membrane potential insensitive to changes in [Na^+^] (31). The channels are regulated by luminal pH [45], PI(3,5)P2 [439], intracellular ATP and extracellular amino acids [46]. TPCs are also involved in the NAADP‐activated Ca^2+^ release from lysosomal Ca^2+^ stores [43, 272]. Mice lacking TPCs are viable but have phenotypes including compromised lysosomal pH stability, reduced physical endurance [46], resistance to Ebola viral infection [358] and fatty liver [124]. No major human disease‐associated TPC mutation has been reported.

### Further reading on CatSper and Two‐Pore channels

Clapham DE et al. (2005) International Union of Pharmacology. L. Nomenclature and structure‐function relationships of CatSper and two‐pore channels. Pharmacol. Rev. 57: 451‐4 [PMID:16382101]

Grimm C et al. (2017) Two‐Pore Channels: Catalyzers of Endolysosomal Transport and Function. Front Pharmacol 8: 45 [PMID:28223936]

Kintzer AF et al. (2017) On the Structure and Mechanism of Two‐Pore Channels. FEBS J [PMID:28656706]

## Cyclic nucleotide‐regulated channels

### Overview

Cyclic nucleotide‐gated (CNG) channels are responsible for signalling in the primary sensory cells of the vertebrate visual and olfactory systems. **A standardised nomenclature for CNG channels has been proposed by the** **NC‐IUPHAR** **subcommittee on voltage‐gated ion channels [** **154** **]**.

CNG channels are voltage‐independent cation channels formed as tetramers. Each subunit has 6TM, with the pore‐forming domain between TM5 and TM6. CNG channels were first found in rod photoreceptors [107, 188], where light signals through rhodopsin and transducin to stimulate phosphodiesterase and reduce intracellular cyclic GMP level. This results in a closure of CNG channels and a reduced ‘dark current’. Similar channels were found in the cilia of olfactory neurons [282] and the pineal gland [95]. The cyclic nucleotides bind to a domain in the C terminus of the subunit protein: other channels directly binding cyclic nucleotides include HCN, eag and certain plant potassium channels.

**Table:** 

Nomenclature	CNGA1	CNGA2	CNGA3	CNGB3
HGNC, UniProt	CNGA1, P29973	CNGA2, Q16280	CNGA3, Q16281	CNGB3, Q9NQW8
Activators	cyclic GMP (EC_50_ ∼30 *μ*M) ≫cyclic AMP	cyclic GMP>cyclic AMP (EC_50_ ∼ 1 *μ*M)	cyclic GMP (EC_50_ ∼ 30 *μ*M) ≫cyclic AMP	–
Inhibitors	–	–	L‐(cis)‐diltiazem (high affinity binding requires presence of CNGB subunits)	–
Channel blockers	dequalinium (pIC_50_ 6.7) [0mV] [355], L‐(cis)‐diltiazem (high affinity binding requires presence of CNGB subunits) (p*K*_i_ 4) [‐80mV – 80mV] [58]	dequalinium (pIC_50_ 5.6) [0mV] [354]	–	L‐(cis)‐diltiazem (Channel blocker when CNGB3 coexpressed with CNGA3) (pIC_50_ 5.5) [0mV] [116] – Mouse
Functional Characteristics	*γ* = 25‐30 pS P_Ca_/P_Na_ = 3.1	*γ* = 35 pS P_Ca_/P_Na_ = 6.8	*γ* = 40 pS P_Ca_/P_Na_ = 10.9	–

### Comments

CNGA1, CNGA2 and CNGA3 express functional channels as homomers. Three additional subunits CNGA4 (Q8IV77), CNGB1 (Q14028) and CNGB3(Q9NQW8) do not, and are referred to as auxiliary subunits. The subunit composition of the native channels is believed to be as follows. Rod: CNGA1_3_/CNGB1a; Cone: CNGA3_2_/CNGB3_2_; Olfactory neurons: CNGA2_2_/CNGA4/CNGB1b [323, 445, 480, 481, 483].

### Hyperpolarisation‐activated, cyclic nucleotide‐gated (HCN) channels

The hyperpolarisation‐activated, cyclic nucleotide‐gated (HCN) channels are cation channels that are activated by hyperpolarisation at voltages negative to ~‐50 mV. The cyclic nucleotides cyclic AMP and cyclic GMP directly activate the channels and shift the activation curves of HCN channels to more positive voltages, thereby enhancing channel activity. HCN channels underlie pacemaker currents found in many excitable cells including cardiac cells and neurons [92, 308]. In native cells, these currents have a variety of names, such as I_h_, I_q_ andI_f_. The four known HCN channels have six transmembrane domains and form tetramers. It is believed that the channels can form heteromers with each other, as has been shown for HCN1 and HCN4 [7]. **A standardised nomenclature for HCN channels has been proposed by the** **NC‐IUPHAR** **subcommittee on voltage‐gated ion channels [** **154** **]**.

**Table:** 

Nomenclature	HCN1	HCN2	HCN3	HCN4
HGNC, UniProt	HCN1, O60741	HCN2, Q9UL51	HCN3, Q9P1Z3	HCN4, Q9Y3Q4
Activators	cyclic AMP>cyclic GMP (both weak)	cyclic AMP>cyclic GMP	–	cyclic AMP>cyclic GMP
Channel blockers	ivabradine (pIC_50_ 5.7) [384], ZD7288 (pIC_50_ 4.7) [383], Cs^+^ (pIC_50_ 3.7) [‐40mV] [383]	ivabradine (pIC_50_ 5.6) [384] – Mouse, ZD7288 (pIC_50_ 4.4) [383], Cs^+^ (pIC_50_ 3.7) [‐40mV] [383]	ivabradine (pIC_50_ 5.7) [384], ZD7288 (pIC_50_ 4.5) [383], Cs^+^ (pIC_50_ 3.8) [‐40mV] [383]	ivabradine (pIC_50_ 5.7) [384], ZD7288 (pIC_50_ 4.7) [383], Cs^+^ (pIC_50_ 3.8) [‐40mV] [383]

### Comments

HCN channels are permeable to both Na^+^ and K^+^ ions, with a Na^+^/K^+^ permeability ratio of about 0.2. Functionally, they differ from each other in terms of time constant of activation with HCN1 the fastest, HCN4 the slowest and HCN2 and HCN3 intermediate. The compounds ZD7288[37] and ivabradine[42] have proven useful in identifying and studying functional HCN channels in native cells. Zatebradine and cilobradine are also useful blocking agents.

### Further reading on Cyclic nucleotide‐regulated channels

Herrmann S et al. (2015) HCN channels–modulators of cardiac and neuronal excitability. Int J Mol Sci 16: 1429‐47 [PMID:25580535]

Hofmann F et al. (2005) International Union of Pharmacology. LI. Nomenclature and structure‐function relationships of cyclic nucleotide‐regulated channels. Pharmacol Rev 57: 455‐62 [PMID:16382102]

Podda MV et al. (2014) New perspectives in cyclic nucleotide‐mediated functions in the CNS: the emerging role of cyclic nucleotide‐gated (CNG) channels. Pflugers Arch 466: 1241‐57 [PMID:24142069]

Tsantoulas C et al. (2016) HCN2 ion channels: basic science opens up possibilities for therapeutic intervention in neuropathic pain. Biochem J 473: 2717‐36 [PMID:27621481]

## Potassium channels

### Overview

Activation of potassium channels regulates excitability and can control the shape of the action potential waveform. They are present in all cells within the body and can influence processes as diverse as cognition, muscle contraction and hormone secretion. Potassium channels are subdivided into families, based on their structural and functional properties. The largest family consists of potassium channels that activated by membrane depolarization, with other families consisting of channels that are either activated by a rise of intracellular calcium ions or are constitutively active. A standardised nomenclature for potassium channels has been proposed by the **NC‐IUPHAR subcommittees** on potassium channels [120, 135, 211, 444], which has placed cloned channels into groups based on gene family and structure of channels that exhibit 6, 4 or 2 transmembrane domains (TM).

## Calcium‐ and sodium‐activated potassium channels

### Overview

The 6TM family of K channels comprises the voltage‐gated K_V_subfamilies, including the KCNQ subfamily, the EAG subfamily (which includes herg channels), the Ca^2+^‐activated Slo subfamily (actually with 6 or 7TM) and the Ca^2+^‐ and Na^+^‐activated SK subfamily **(nomenclature as agreed by the NC‐IUPHAR Subcommittee on Calcium‐ and sodium‐activated potassium channels [** **181** **])**. As for the 2TM family, the pore‐forming a subunits form tetramers and heteromeric channels may be formed within subfamilies (e.g. K_V_1.1 with K_V_1.2; KCNQ2 with KCNQ3).

**Table:** 

Nomenclature	K_Ca_1.1	K_Ca_2.1	K_Ca_2.2	K_Ca_2.3	K_Ca_3.1
HGNC, UniProt	KCNMA1, Q12791	KCNN1, Q92952	KCNN2, Q9H2S1	KCNN3, Q9UGI6	KCNN4, O15554
Activators	NS004, NS1619	EBIO Concentration range: 2×10^−3^M [‐80mV] [320, 442], NS309 Concentration range: 3×10^−8^M‐1×10^−7^M [‐90mV] [388, 442]	NS309 (pEC_50_ 6.2) Concentration range: 3×10^−8^M‐1×10^−7^M [319, 388, 442], EBIO (pEC_50_ 3.3) [319, 442], EBIO (pEC_50_ 3) Concentration range: 2×10^−3^M [48, 320] – Rat	EBIO (pEC_50_ 3.8) [442, 450], NS309 Concentration range: 3×10^−8^M [388, 442]	NS309 (pEC_50_ 8) [‐90mV] [388, 442], SKA‐121 (pEC_50_ 7) [72], EBIO (pEC_50_ 4.1–4.5) [‐100mV – ‐50mV] [320, 394, 442]
Inhibitors	paxilline (p*K*_i_ 8.7) [0mV] [360] – Mouse	UCL1684 (pIC_50_ 9.1) [387, 442], apamin (pIC_50_ 7.9–8.5) [367, 385, 387]	UCL1684 (pIC_50_ 9.6) [103, 442], apamin (p*K*_d_ 9.4) [180]	apamin (pIC_50_ 7.9–9.1) [407, 450], UCL1684 (pIC_50_ 8–9) [103, 442]	TRAM‐34 (p*K*_d_ 7.6–8) [213, 456]
Channel blockers	charybdotoxin, iberiotoxin, tetraethylammonium	tetraethylammonium (pIC_50_ 2.7) [442]	tetraethylammonium (pIC_50_ 2.7) [442]	tetraethylammonium (pIC_50_ 2.7) [442]	charybdotoxin (pIC_50_ 7.6–8.7) [171, 176]
Functional Characteristics	Maxi K_Ca_	SK_Ca_	SK_Ca_	SK_Ca_	IK_Ca_
Comments	–	The rat isoform does not form functional channels when expressed alone in cell lines. N‐ or C‐terminal chimeric constructs permit functional channels that are insensitive to apamin [442]. Heteromeric channels are formed between K_Ca_2.1 and 2.2 subunits that show intermediate sensitivity to apamin [68].	–	–	–

**Table:** 

Nomenclature	K_Na_1.1	K_Na_1.2	K_Ca_5.1
HGNC, UniProt	KCNT1, Q5JUK3	KCNT2, Q6UVM3	KCNU1, A8MYU2
Activators	bithionol (pEC_50_ 5–6) [470] – Rat, niclosamide (pEC_50_ 5.5) [32], loxapine (pEC_50_ 5.4) [32]	niflumic acid (pEC_50_ 8.7) [78, 115]	–
Gating inhibitors	bepridil (pIC_50_ 5–6) [470] – Rat	–	–
Channel blockers	quinidine (pIC_50_ 4) [29, 470] – Rat	Ba^2+^ (pIC_50_ 3) [29], quinidine Concentration range: 1×10^−3^M [29] – Rat	quinidine Concentration range: 2×10^−5^M [404, 454] – Mouse
Functional Characteristics	K_Na_	K_Na_	Sperm pH‐regulated K^+^ current, KSPER

## Inwardly rectifying potassium channels

### Overview

The 2TM domain family of K channels are also known as the inward‐rectifier K channel family. This family includes the strong inward‐rectifier K channels (K_ir_2.x) that are constitutively active, the G‐protein‐activated inward‐rectifier K channels (K_ir_3.x) and the ATP‐sensitive K channels (K_ir_6.x, which combine with sulphonylurea receptors (SUR1‐3)). The pore‐forming *α* subunits form tetramers, and heteromeric channels may be formed within subfamilies (e.g. K_ir_3.2 with K_ir_3.3).

**Table:** 

Nomenclature	K_ir_1.1
HGNC, UniProt	KCNJ1, P48048
Ion Selectivity and Conductance	NH_4_^+^ [62pS] > K^+^ [38. pS] > Tl^+^ [21pS] > Rb^+^ [15pS] (Rat) [62, 150]
Channel blockers	tertiapin‐Q (pIC_50_ 8.9) [175], Ba^2+^ (pIC_50_ 2.3–4.2) Concentration range: 1×10^−4^M [voltage dependent 0mV – ‐100mV] [150, 484] – Rat, Cs^+^ (pIC_50_ 2.9) [voltage dependent ‐120mV] [484] – Rat
Functional Characteristics	K_ir_1.1 is weakly inwardly rectifying, as compared to classical (strong) inward rectifiers.

**Table:** 

Nomenclature	K_ir_1.1	K_ir_2.1	K_ir_2.2	K_ir_2.3
HGNC, UniProt	KCNJ2, P63252	KCNJ12, Q14500	KCNJ4, P48050	KCNJ14, Q9UNX9
Endogenous activators	PIP_2_ Concentration range: 1×10^−5^M‐5×10^−5^M [‐30mV] [158, 348, 379] – Mouse	–	–	–
Endogenous inhibitors	–	Intracellular Mg^2+^ (pIC_50_ 5) [40mV] [469]	–	Intracellular Mg^2+^
Gating inhibitors	–	Ba^2+^ Concentration range: 5×10^−5^M [‐150mV – ‐50mV] [397] – Mouse, Cs^+^ Concentration range: 5×10^−6^M‐5×10^−5^M [‐150mV – ‐50mV] [397] – Mouse	–	–
Endogenous channel blockers	spermine (p*K*_d_ 9.1) [voltage dependent 40mV] [167, 471] – Mouse, spermidine (p*K*_d_ 8.1) [voltage dependent 40mV] [471] – Mouse, putrescine (p*K*_d_ 5.1) [voltage dependent 40mV] [167, 471] – Mouse, Intracellular Mg^2+^ (p*K*_d_ 4.8) [voltage dependent 40mV] [471] – Mouse	–	Intracellular Mg^2+^ (p*K*_d_ 5) [voltage dependent 50mV] [246], putrescine Concentration range: 5×10^−5^M‐1×10^−3^M [‐80mV – 80mV] [246], spermidine Concentration range: 2.5×10^−5^M‐1×10^−3^M [‐80mV – 80mV] [246], spermine Concentration range: 5×10^−5^M‐1×10^−3^M [‐80mV – 80mV] [246]	
Channel blockers	Ba^2+^ (p*K*_d_ 3.9–5.6) Concentration range: 1×10^−6^M‐1×10^−4^M [voltage dependent 0mV – ‐80mV] [6] – Mouse, Cs^+^ (p*K*_d_ 1.3–4) Concentration range: 3×10^−5^M‐3×10^−4^M [voltage dependent 0mV – ‐102mV] [3] – Mouse	–	Ba^2+^ (pIC_50_ 5) Concentration range: 3×10^−6^M‐5×10^−4^M [‐60mV] [260, 335, 405], Cs^+^ (p*K*_i_ 1.3–4.5) Concentration range: 3×10^−6^M‐3×10^−4^M [0mV – ‐130mV] [260]	Cs^+^ (p*K*_d_ 3–4.1) [voltage dependent ‐100mV – ‐60mV] [159], Ba^2+^ (p*K*_d_ 3.3) [voltage dependent 0mV] [159]
Functional Characteristics	IK_1_ in heart, ‘strong’ inward–rectifier current	IK_1_in heart, ‘strong’ inward–rectifier current	IK_1_ in heart, ‘strong’ inward–rectifier current	IK_1_ in heart, ‘strong’ inward–rectifier current
Comments	K_ir_2.1 is also inhibited by intracellular polyamines	K_ir_2.2 is also inhibited by intracellular polyamines	K_ir_2.3 is also inhibited by intracellular polyamines	K_ir_2.4 is also inhibited by intracellular polyamines

**Table:** 

Nomenclature	K_ir_3.1	K_ir_3.2	K_ir_3.3	K_ir_3.4
HGNC, UniProt	KCNJ3, P48549	KCNJ6, P48051	KCNJ9, Q92806	KCNJ5, P48544
Endogenous activators	PIP_2_ (p*K*_d_ 6.3) Concentration range: 5×10^−5^M [physiological voltage] [158]	PIP_2_ (p*K*_d_ 6.3) Concentration range: 5×10^−5^M [physiological voltage] [158]	PIP_2_ [145]	PIP_2_ [20, 145]
Gating inhibitors	–	pimozide (Data obtained using K_ir_3.1/3.2 heteromer) (pEC_50_ 5.5) [‐70mV] [201] – Mouse	–	–
Channel blockers	tertiapin‐Q (K_ir_3.1/3.4; expression in Xenopus oocytes) (pIC_50_ 7.9) [174], Ba^2+^ (K_ir_3.1 expressed in Xenopus oocytes) (pIC_50_ 4.7) [80] – Rat	desipramine (Data obtained using K_ir_3.1/3.2 heteromer) (pIC_50_ 4.4) [‐70mV] [202] – Mouse	–	tertiapin‐Q (K_ir_3.1/3.4) (pIC_50_ 7.9) [174]
Functional Characteristics	G protein‐activated inward‐rectifier current	G protein‐activated inward‐rectifier current	G protein‐activated inward‐rectifier current	G protein‐activated inward‐rectifier current
Comments	K_ir_3.1 is also activated by G_*βγ*_. K_ir_3.1 is not functional alone. The functional expression of K_ir_3.1 in Xenopus oocytes requires coassembly with the endogenous Xenopus K_ir_3.5 subunit. The major functional assembly in the heart is the K_ir_3.1/3.4 heteromultimer, while in the brain it is K_ir_3.1/3.2, K_ir_3.1/3.3 and K_ir_3.2/3.3.	K_ir_3.2 is also activated by G_*βγ*_. K_ir_3.2 forms functional heteromers with K_ir_3.1/3.3.	K_ir_3.3 is also activated by G_*βγ*_	K_ir_3.4 is also activated by G_*βγ*_

**Table:** 

Nomenclature	K_ir_4.1	K_ir_4.2	K_ir_5.1
HGNC, UniProt	KCNJ10, P78508	KCNJ15, Q99712	KCNJ16, Q9NPI9
Channel blockers	Ba^2+^ Concentration range: 3×10^−6^M‐1×10^−3^M [‐160mV – 60mV] [205, 399, 403] – Rat, Cs^+^ Concentration range: 3×10^−5^M‐3×10^−4^M [‐160mV – 50mV] [399] – Rat	Ba^2+^ (K_ir_4.2 expressed in Xenopus oocytes) Concentration range: 1×10^−5^M‐1×10^−4^M [‐120mV – 100mV] [318] – Mouse, Cs^+^ (K_ir_4.2 expressed in Xenopus oocytes) Concentration range: 1×10^−5^M‐1×10^−4^M [‐120mV – 100mV] [318] – Mouse	Ba^2+^ (K_ir_5.1 expressed with PSD‐95) Concentration range: 3×10^−3^M [‐120mV – 20mV] [402] – Rat
Functional Characteristics	Inward‐rectifier current	Inward‐rectifier current	Weakly inwardly rectifying

**Table:** 

Nomenclature	K_ir_6.1	K_ir_6.2	K_ir_7.1
HGNC, UniProt	KCNJ8, Q15842	KCNJ11, Q14654	KCNJ13, O60928
Associated subunits	SUR1, SUR2A, SUR2B	SUR1, SUR2A, SUR2B	–
Activators	cromakalim, diazoxide Concentration range: 2×10^−4^M [‐60mV] [466] – Mouse, minoxidil, nicorandil Concentration range: 3×10^−4^M [‐60mV – 60mV] [466] – Mouse	diazoxide (pEC_50_ 4.2) [physiological voltage] [162] – Mouse, cromakalim Concentration range: 3×10^−5^M [‐60mV] [163] – Mouse, minoxidil, nicorandil	–
Inhibitors	glibenclamide, tolbutamide	glibenclamide, tolbutamide	–
Channel blockers	–	–	Ba^2+^ (p*K*_i_ 3.2) [voltage dependent ‐100mV] [99, 210, 212, 311], Cs^+^ (p*K*_i_ 1.6) [voltage dependent ‐100mV] [99, 210, 311]
Functional Characteristics	ATP‐sensitive, inward‐rectifier current	ATP‐sensitive, inward‐rectifier current	Inward‐rectifier current

## Two P domain potassium channels

### Overview

The 4TM family of K channels mediate many of the background potassium currents observed in native cells. They are open across the physiological voltage‐range and are regulated by a wide array of neurotransmitters and biochemical mediators. The pore‐forming *α*‐subunit contains two pore loop (P) domains and two subunits assemble to form one ion conduction pathway lined by four P domains. It is important to note that single channels do not have two pores but that each subunit has two P domains in its primary sequence; hence the name two P domain, or K_2P_ channels (and not two‐pore channels). Some of the K_2P_ subunits can form heterodimers across subfamilies (e.g. K_2P_3.1 with K_2P_9.1). The nomenclature of 4TM K channels in the literature is still a mixture of IUPHAR and common names. The suggested division into subfamilies, below, is based on similarities in both structural and functional properties within subfamilies.

**Table:** 

Nomenclature	K_2P_1.1	K_2P_2.1	K_2P_3.1	K_2P_4.1
HGNC, UniProt	KCNK1, O00180	KCNK2, O95069	KCNK3, O14649	KCNK4, Q9NYG8
Endogenous activators	–	arachidonic acid (studied at 1‐10 μM) (pEC_50_ 5) [314]	–	arachidonic acid (studied at 1‐10 μM) [108]
Activators	–	chloroform (studied at 1‐5 mM) Concentration range: 8×10^−3^M [313], halothane (studied at 1‐5 mM) [313], isoflurane (studied at 1‐5 mM) [313]	halothane (studied at 1‐10 mM)	riluzole (studied at 1‐100 μM) [97]
Channel blockers	–	–	R‐(+)‐methanandamide (pIC_50_∼6.2) [257], anandamide (pIC_50_∼6.2) [257]	–
Functional Characteristics	Background current	Background current	Background current	Background current
Comments	K_2P_1.1 is inhibited by acid pH_o_ external acidification with a pK_a_ ∼ 6.7 [331]. K_2P_1 forms heterodimers with K_2P_3 and K_2P_9 [332].	K_2P_2.1 is also activated by membrane stretch, heat and acid pH_i_ [256, 258]. K_2P_2 can heterodimerize with K_2P_4 [33] and K_2P_10 [228].	Knock‐out of the kcnk3 gene leads to a prolonged QT interval in mice [83] and disrupted development of the adrenal cortex [143]. K_2P_3.1 is inhibited by acid pH_o_ with a pK_a_ of 6.4 [247]. K_2P_3 forms heterodimers with K_2P_1 [332] and K_2P_9 [77].	K_2P_4 is activated by membrane stretch [255], and increased temperature ( ∼ 12 to 20‐fold between 17 and 40°C [183]) and can heterodimerize with K_2P_2 [33].

**Table:** 

Nomenclature	K_2P_5.1	K_2P_6.1	K_2P_7.1	K_2P_9.1
HGNC, UniProt	KCNK5, O95279	KCNK6, Q9Y257	KCNK7, Q9Y2U2	KCNK9, Q9NPC2
Activators	–	–	–	halothane (studied at 1‐5 mM) [401]
Inhibitors	–	–	–	R‐(+)‐methanandamide (studied at 1‐10 μM) [343], anandamide (studied at 1‐10 μM) [343]
Functional Characteristics	Background current	Unknown	Unknown	Background current
Comments	K_2P_5.1 is activated by alkaline pH_o_ [351]. Knockout of the kcnk5 gene in mice is associated with metabolic acidosis, hyponatremia and hypotension due to impaired bicarbonate handling in the kidney [441], as well as deafness [55]. The T108P mutation is associated with Balkan Endemic Nephropathy in humans [414].	–	–	K_2P_9.1 is also inhibited by acid pH_o_ with a pK_a_ of ∼ 6 [343]. Imprinting of the KCNK9 gene is associated with Birk Barel syndrome [18]. K_2P_9 can form heterodimers with K_2P_1 [332] or K_2P_3 [77].

**Table:** 

Nomenclature	K_2P_10.1	K_2P_12.1	K_2P_13.1	K_2P_15.1	K_2P_16.1	K_2P_17.1	K_2P_18.1
HGNC, UniProt	KCNK10, P57789	KCNK12, Q9HB15	KCNK13, Q9HB14	KCNK15, Q9H427	KCNK16, Q96T55	KCNK17, Q96T54	KCNK18, Q7Z418
Endogenous activators	arachidonic acid (studied at 1‐10 μM) [225]	–	–	–	–	–	–
Activators	halothane (studied at 1‐5 mM) [225]	–	–	–	–	–	–
Endogenous inhibitors	–	–	–	–	–	–	arachidonic acid (studied at 10‐50 μM) [361]
Inhibitors	norfluoxetine (pIC_50_ 5.1) [189]	–	halothane (studied at ∼ 5 mM) [34]	–	–	–	–
Functional Characteristics	Background current	Does not function as a homodimer [342] but can form a functional heterodimer with K_2P_13 [34].	Background current	Unknown	Background current	Background current	Background current
Comments	K_2P_10.1 is also activated by membrane stretch [225] and can heterodimerize with K_2P_2 [228].	–	Forms a heterodimer with K_2P_12 [34].	–	K_2P_16.1 current is increased by alkaline pH_o_ with a pK_a_ of 7.8 [184].	K_2P_17.1 current is increased by alkaline pH_o_ with a pK_a_ of 8.8 [184].	A frame‐shift mutation (F139WfsX24) in the KCNK18 gene, is associated with migraine with aura in humans [214].

### Comments

The K_2P_6, K_2P_7.1, K_2P_15.1 and K_2P_12.1 subtypes, when expressed in isolation, are nonfunctional. All 4TM channels are insensitive to the classical potassium channel blockers tetraethylammonium and fampridine, but are blocked to varying degrees by Ba^2+^ ions.

## Voltage‐gated potassium channels

### Overview

The 6TM family of K channels comprises the voltage‐gated K_V_subfamilies, the EAG subfamily (which includes hERG channels), the Ca^2+^‐activated Slo subfamily (actually with 7TM, termed BK) and the Ca^2+^‐activated SK subfamily. These channels possess a pore‐forming *α* subunit that comprise tetramers of identical subunits (homomeric) or of different subunits (heteromeric). Heteromeric channels can only be formed within subfamilies (e.g. K_v_1.1 with K_v_1.2; K_v_7.2 with K_v_7.3). The pharmacology largely reflects the subunit composition of the functional channel.

**Table:** 

Nomenclature	K_v_1.1	K_v_1.2	K_v_1.3	K_v_1.4
HGNC, UniProt	KCNA1, Q09470	KCNA2, P16389	KCNA3, P22001	KCNA4, P22459
Associated subunits	K_v_1.2, K_v_1.4, K_v_ *β*1 and K_v_ *β*2 [73]	K_v_1.1, K_v_1.4, K_v_ *β*1 and K_v_ *β*2 [73]	K_v_1.1, K_v_1.2, K_v_1.4, K_v_1.6 , K_v_ *β*1 and K_v_ *β*2 [73]	K_v_1.1, K_v_1.2, K_v_ *β*1 and K_v_ *β*2 [73]
Channel blockers	*α*‐dendrotoxin (pEC_50_ 7.7–9) [128, 160] – Rat, margatoxin (pIC_50_ 8.4) [19], tetraethylammonium (p*K*_d_ 3.5) [128] – Mouse	margatoxin (pIC_50_ 11.2) [19], *α*‐dendrotoxin (pIC_50_ 7.8–9.4) [128, 160] – Rat, noxiustoxin (p*K*_d_ 8.7) [128] – Rat	margatoxin (pIC_50_ 10–10.3) [113, 117], noxiustoxin (p*K*_d_ 9) [128] – Mouse, maurotoxin (pIC_50_ 6.8) [352], tetraethylammonium (p*K*_d_ 2) [128] – Mouse	fampridine (pIC_50_ 1.9) [391] – Rat
Selective channel blockers	–	–	correolide (pIC_50_ 7.1) [106]	–
Functional Characteristics	K_V_	K_V_	K_V_	K_A_
Comments	–	–	Resistant to dendrotoxins	Resistant to dendrotoxins

**Table:** 

Nomenclature	K_v_1.5	K_v_1.6	K_v_1.7	K_v_1.8
HGNC, UniProt	KCNA5, P22460	KCNA6, P17658	KCNA7, Q96RP8	KCNA10, Q16322
Associated subunits	K_v_ *β*1 and K_v_ *β*2	K_v_ *β*1 and K_v_ *β*2	K_v_ *β*1 and K_v_ *β*2	K_v_ *β*1 and K_v_ *β*2
Channel blockers	fampridine (pIC_50_ 4.3) [105]	*α*‐dendrotoxin (pIC_50_ 7.7) [129], tetraethylammonium (pIC_50_ 2.2) [129]	noxiustoxin (pIC_50_ 7.7) [182] – Mouse, fampridine (pIC_50_ 3.6) [182] – Mouse	fampridine (pIC_50_ 2.8) [217]
Functional Characteristics	K_v_	K_V_	K_V_	K_V_
Comments	Resistant to external TEA	–	–	–

**Table:** 

Nomenclature	K_v_2.1	K_v_2.2	K_v_3.1	K_v_3.2	K_v_3.3	K_v_3.4
HGNC, UniProt	KCNB1, Q14721	KCNB2, Q92953	KCNC1, P48547	KCNC2, Q96PR1	KCNC3, Q14003	KCNC4, Q03721
Associated subunits	K_v_5.1, K_v_6.1‐6.4, K_v_8.1‐8.2 and K_v_9.1‐9.3	K_v_5.1, K_v_6.1‐6.4, K_v_8.1‐8.2 and K_v_9.1‐9.3	–	–	–	MiRP2 is an associated subunit for K_v_3.4
Channel blockers	tetraethylammonium (pIC_50_ 2) [142] – Rat	fampridine (pIC_50_ 2.8) [363], tetraethylammonium (pIC_50_ 2.6) [363]	fampridine (pIC_50_ 4.5) [128] – Mouse, tetraethylammonium (pIC_50_ 3.7) [128] – Mouse	fampridine (pIC_50_ 4.6) [233] – Rat, tetraethylammonium (pIC_50_ 4.2) [233] – Rat	tetraethylammo‐ nium (pIC_50_ 3.9) [419] – Rat	tetraethylammonium (pIC_50_ 3.5) [350, 365] – Rat
Selective channel blockers	–	–	–	–	–	sea anemone toxin BDS‐I (pIC_50_ 7.3) [93] – Rat
Functional Characteristics	K_V_	–	K_V_	K_V_	K_A_	K_A_

**Table:** 

Nomenclature	K_v_4.1	K_v_4.2	K_v_4.3
HGNC, UniProt	KCND1, Q9NSA2	KCND2, Q9NZV8	KCND3, Q9UK17
Associated subunits	KChIP 1‐4, DP66, DPP10	KChIP 1‐4, DPP6, DPP10, K_v_ *β*1, NCS‐1, Na_v_ *β*1	KChIP 1‐4, DPP6 and DPP10, MinK, MiRPs
Channel blockers	fampridine (pIC_50_ 2) [166]	–	–
Functional Characteristics	K_A_	K_A_	K_A_

**Table:** 

Nomenclature	K_v_5.1	K_v_6.1	K_v_6.2	K_v_6.3	K_v_6.4
HGNC, UniProt	KCNF1, Q9H3M0	KCNG1, Q9UIX4	KCNG2, Q9UJ96	KCNG3, Q8TAE7	KCNG4, Q8TDN1

**Table:** 

Nomenclature	K_v_7.1	K_v_7.2	K_v_7.3	K_v_7.4	K_v_7.5
HGNC, UniProt	KCNQ1, P51787	KCNQ2, O43526	KCNQ3, O43525	KCNQ4, P56696	KCNQ5, Q9NR82
Activators	–	retigabine (pEC_50_ 5.6) [406]	retigabine (pEC_50_ 6.2) [406]	retigabine (pEC_50_ 5.2) [406]	retigabine (pEC_50_ 5) [98]
Inhibitors	XE991 (p*K*_d_ 6.1) [436], linopirdine (pIC_50_ 4.4) [302] – Mouse	XE991 (pIC_50_ 6.2) [437], linopirdine (pIC_50_ 5.3) [437],	linopirdine (pIC_50_ 5.4) [437] – Rat	XE991 (pIC_50_ 5.3) [396], linopirdine (pIC_50_ 4.9) [396],	linopirdine (p*K*_d_ 4.8) [224], XE991 (pIC_50_ 4.2) [364]
Channel blockers		tetraethylammonium (pIC_50_ 3.5–3.9) [136, 446]	–	tetraethylammonium (pIC_50_ 1.3) [13]	
Functional Characteristics	cardiac IK_5_	M current as a heteromer between K_V_7.2 and K_V_7.3	M current as heteromeric K_V_7.2/K_V_7.3 or K_V_7.3/K_V_7.5	–	M current as heteromeric K_V_7.3/K_V_7.5

**Table:** 

Nomenclature	K_v_8.1	K_v_8.2	K_v_9.1	K_v_9.2	K_v_9.3	K_v_10.1	K_v_10.2
HGNC, UniProt	KCNV1, Q6PIU1	KCNV2, Q8TDN2	KCNS1, Q96KK3	KCNS2, Q9ULS6	KCNS3, Q9BQ31	KCNH1, O95259	KCNH5, Q8NCM2

**Table:** 

Nomenclature	K_v_11.1	K_v_11.2	K_v_11.3	K_v_12.1	K_v_12.2	K_v_12.3
HGNC, UniProt	KCNH2, Q12809	KCNH6, Q9H252	KCNH7, Q9NS40	KCNH8, Q96L42	KCNH3, Q9ULD8	KCNH4, Q9UQ05
Associated subunits	minK (KCNE1) and MiRP1 (KCNE2)	minK (KCNE1)	minK (KCNE1)	minK (KCNE1)	minK (KCNE1) and MiRP2 (KCNE3)	–
Channel blockers	astemizole (pIC_50_ 9) [486], terfenadine (pIC_50_ 7.3) [344], disopyramide (pIC_50_ 4) [190]	–	–	–	–	–
Inhibitor	E4031 (pIC_50_ 8.1) [485]	–	–	–	–	–
Selective channel blockers	dofetilide (p*K*_i_ 8.2) [372], ibutilide (pIC_50_ 7.6–8) [190, 326]	–	–	–	–	–
Functional Characteristics	cardiac IK_R_	–	–	–	–	–
Comments	RPR260243 is an activator of K_v_11.1 [185].	–	–	–	–	–

### Further reading on Potassium channels

Chang PC et al. (2015) SK channels and ventricular arrhythmias in heart failure. Trends Cardiovasc Med 25: 508‐14 [PMID:25743622]

Decher N et al. (2017) Stretch‐activated potassium currents in the heart: Focus on TREK‐1 and arrhythmias. Prog Biophys Mol Biol [PMID:28526352]

Feliciangeli S et al. (2015) The family of K2P channels: salient structural and functional properties. J Physiol 593: 2587‐603 [PMID:25530075]

Foster MN et al. (2016) KATP Channels in the Cardiovascular System. Physiol Rev 96: 177‐252 [PMID:26660852]

Goldstein SA et al. (2005) International Union of Pharmacology. LV. Nomenclature and molecular relationships of two‐P potassium channels. Pharmacol Rev 57: 527‐40 [PMID:16382106]

Greene DL et al. (2017) Modulation of Kv7 channels and excitability in the brain. Cell Mol Life Sci 74: 495‐508 [PMID:27645822]

Gutman GA et al. (2003) International Union of Pharmacology. XLI. Compendium of voltage‐gated ion channels: potassium channels. Pharmacol Rev 55: 583‐6 [PMID:14657415]

Kaczmarek LK et al. (2017) International Union of Basic and Clinical Pharmacology. C. Nomenclature and Properties of Calcium‐Activated and Sodium‐Activated Potassium Channels. Pharmacol Rev 69: 1‐11 [PMID:28267675]

Kubo Y et al. (2005) International Union of Pharmacology. LIV. Nomenclature and molecular relationships of inwardly rectifying potassium channels. Pharmacol Rev 57: 509‐26 [PMID:16382105]

Latorre R et al. (2017) Molecular Determinants of BK Channel Functional Diversity and Functioning. Physiol Rev 97: 39‐87 [PMID:27807200]

Niemeyer MI et al. (2016) Gating, Regulation, and Structure in K2P K+ Channels: In Varietate Concordia? Mol Pharmacol 90: 309‐17 [PMID:27268784]

Poveda JA et al. (2017) Towards understanding the molecular basis of ion channel modulation by lipids: Mechanistic models and current paradigms. Biochim Biophys Acta 1859: 1507‐1516 [PMID:28408206]

Rifkin RA et al. (2017) G Protein‐Gated Potassium Channels: A Link to Drug Addiction. Trends Pharmacol Sci 38: 378‐392 [PMID:28188005]

Taylor KC et al. (2017) Regulation of KCNQ/Kv7 family voltage‐gated K+ channels by lipids. Biochim Biophys Acta 1859: 586‐597 [PMID:27818172]

Vivier D et al. (2016) Perspectives on the Two‐Pore Domain Potassium Channel TREK‐1 (TWIK‐Related K(+) Channel 1). A Novel Therapeutic Target? J Med Chem 59: 5149‐57 [PMID:26588045]

Wei AD et al. (2005) International Union of Pharmacology. LII. Nomenclature and molecular relationships of calcium‐activated potassium channels. Pharmacol Rev 57: 463‐72 [PMID:16382103]

Yang KC et al. (2016) Mechanisms contributing to myocardial potassium channel diversity, regulation and remodeling. Trends Cardiovasc Med 26: 209‐18 [PMID:26391345]

Grissmer M et al. (2005) International Union of Pharmacology. LIII. Nomenclature and molecular relationships of voltage‐gated potassium channels. Pharmacol Rev 57: 473‐508 [PMID:16382104]

## Ryanodine receptors

### Overview

The ryanodine receptors (RyRs) are found on intracellular Ca^2+^ storage/release organelles. The family of RyR genes encodes three highly related Ca^2+^ release channels: RyR1, RyR2 and RyR3, which assemble as large tetrameric structures. These RyR channels are ubiquitously expressed in many types of cells and participate in a variety of important Ca^2+^ signaling phenomena (neurotransmission, secretion, etc.). In addition to the three mammalian isoforms described below, various nonmammalian isoforms of the ryanodine receptor have been identified [392]. The function of the ryanodine receptor channels may also be influenced by closely associated proteins such as the tacrolimus (FK506)‐binding protein, calmodulin [467], triadin, calsequestrin, junctin and sorcin, and by protein kinases and phosphatases.

**Table:** 

Nomenclature	RyR1	RyR2	RyR3
HGNC, UniProt	RYR1, P21817	RYR2, Q92736	RYR3, Q15413
Endogenous activators	cytosolic ATP (endogenous; mM range), cytosolic Ca^2+^ (endogenous; *μ*M range), luminal Ca^2+^ (endogenous)	cytosolic ATP (endogenous; mM range), cytosolic Ca^2+^ (endogenous; *μ*M range), luminal Ca^2+^ (endogenous)	cytosolic ATP (endogenous; mM range), cytosolic Ca^2+^ (endogenous; *μ*M range)
Activators	caffeine (pharmacological; mM range), ryanodine (pharmacological; nM ‐ *μ*M range), suramin (pharmacological; *μ*M range)	caffeine (pharmacological; mM range), ryanodine (pharmacological; nM ‐ *μ*M range), suramin (pharmacological; *μ*M range)	caffeine (pharmacological; mM range), ryanodine (pharmacological; nM ‐ *μ*M range)
Endogenous antagonists	cytosolic Ca^2+^ Concentration range: >1×10^−4^M, cytosolic Mg^2+^ (mM range)	cytosolic Ca^2+^ Concentration range: >1×10^−3^M, cytosolic Mg^2+^ (mM range)	cytosolic Ca^2+^ Concentration range: >1×10^−3^M, cytosolic Mg^2+^ (mM range)
Antagonists	dantrolene	–	dantrolene
Channel blockers	procaine, ruthenium red, ryanodine Concentration range: >1×10^−4^M	procaine, ruthenium red, ryanodine Concentration range: >1×10^−4^M	ruthenium red
Functional Characteristics	Ca^2+^: (P _Ca_/P _K_ ∼6) single‐channel conductance: 90 pS (50mM Ca^2+^), 770 pS (200 mM K^+^)	Ca^2+^: (P _Ca_/P _K_6) single‐channel conductance: 90 pS (50mM Ca^2+^), 720 pS (210 mM K^+^)	Ca^2+^: (P _Ca_/P_K_ 6) single‐channel conductance: 140 pS (50mM Ca^2+^), 777 pS (250 mM K^+^)
Comments	RyR1 is also activated by depolarisation via DHP receptor, calmodulin at low cytosolic Ca^2+^ concentrations, CaM kinase and PKA; antagonised by calmodulin at high cytosolic Ca^2+^ concentrations	RyR2 is also activated by CaM kinase and PKA; antagonised by calmodulin at high cytosolic Ca^2+^ concentrations	RyR3 is also activated by calmodulin at low cytosolic Ca^2+^ concentrations; antagonised by calmodulin at high cytosolic Ca^2+^ concentrations

### Comments

The modulators of channel function included in this table are those most commonly used to identify ryanodine‐sensitive Ca^2+^ release pathways. Numerous other modulators of ryanodine receptor/channel function can be found in the reviews listed below. The absence of a modulator of a particular isoform of receptor indicates that the action of that modulator has not been determined, not that it is without effect. The potential role of cyclic ADP ribose as an endogenous regulator of ryanodine receptor channels is controversial. A region of RyR likely to be involved in ion translocation and selection has been identified [112, 479].

### Further reading on Ryanodine receptors

O'Brien F et al. (2015) The ryanodine receptor provides high throughput Ca2+‐release but is precisely regulated by networks of associated proteins: a focus on proteins relevant to phosphorylation. Biochem Soc Trans 43: 426‐33 [PMID:26009186]

Samso M. (2017) A guide to the 3D structure of the ryanodine receptor type 1 by cryoEM. Protein Sci 26: 52‐68 [PMID:27671094]

Van Petegem F. (2015) Ryanodine receptors: allosteric ion channel giants. J Mol Biol 427: 31‐53 [PMID:25134758]

Zalk R et al. (2017) Ca2+ Release Channels Join the 'Resolution Revolution'. Trends Biochem Sci 42: 543‐555 [PMID:28499500]

## Transient Receptor Potential channels

### Overview

The TRP superfamily of channels (**nomenclature as agreed by** **NC‐IUPHAR** **[** **70** **,** **455** **]**), whose founder member is the Drosophila Trp channel, exists in mammals as six families; TRPC, TRPM, TRPV, TRPA, TRPP and TRPML based on amino acid homologies. TRP subunits contain six putative transmembrane domains and assemble as homo‐ or hetero‐tetramers to form cation selective channels with diverse modes of activation and varied permeation properties (reviewed by [307]). Established, or potential, physiological functions of the individual members of the TRP families are discussed in detail in the recommended reviews and a compilation edited by Islam [168]. The established, or potential, involvement of TRP channels in disease is reviewed in [196, 288] and [290], together with a special edition of Biochemica et Biophysica Acta on the subject [288]. The pharmacology of most TRP channels is poorly developed [455]. Broad spectrum agents are listed in the tables along with more selective, or recently recognised, ligands that are flagged by the inclusion of a primary reference. Most TRP channels are regulated by phosphoinostides such as PtIns(4,5)P_2_and IP_3_ although the effects reported are often complex, occasionally contradictory, and likely to be dependent upon experimental conditions, such as intracellular ATP levels (reviewed by [291, 353, 424]). Such regulation is generally not included in the tables.When thermosensitivity is mentioned, it refers specifically to a high Q10 of gating, often in the range of 10‐30, but does not necessarily imply that the channel's function is to act as a 'hot' or 'cold' sensor. In general, the search for TRP activators has led to many claims for temperature sensing, mechanosensation, and lipid sensing. All proteins are of course sensitive to energies of binding, mechanical force, and temperature, but the issue is whether the proposed input is within a physiologically relevant range resulting in a response.

### TRPA (ankyrin) family

TRPA1 is the sole mammalian member of this group (reviewed by [114]). TRPA1 activation of sensory neurons contribute to nociception [177, 266, 386]. Pungent chemicals such as mustard oil (AITC), allicin, and cinnamaldehyde activate TRPA1 by modification of free thiol groups of cysteine side chains, especially those located in its amino terminus [22, 149, 251, 253]. Alkenals with *α*, *β*‐unsaturated bonds, such as propenal (acrolein), butenal (crotylaldehyde), and 2‐pentenal can react with free thiols via Michael addition and can activate TRPA1. However, potency appears to weaken as carbon chain length increases [11, 22]. Covalent modification leads to sustained activation of TRPA1. Chemicals including carvacrol, menthol, and local anesthetics reversibly activate TRPA1 by non‐covalent binding [186, 222, 460, 461]. TRPA1 is not mechanosensitive under physiological conditions, but can be activated by cold temperatures [86, 187]. The electron cryo‐EM structure of TRPA1 [315] indicates that it is a 6‐TM homotetramer. Each subunit of the channel contains two short ‘pore helices’ pointing into the ion selectivity filter, which is big enough to allow permeation of partially hydrated Ca^2+^ ions. A coiled‐coil domain in the carboxy‐terminal region forms the cytoplasmic stalk of the channel, and is surrounded by 16 ankyrin repeat domains, which are speculated to interdigitate with an overlying helix‐turn‐helix and putative *β*‐sheet domain containing cysteine residues targeted by electrophilic TRPA1 agonists. The TRP domain, a helix at the base of S6, runs perpendicular to the pore helices suspended above the ankyrin repeats below, where it may contribute to regulation of the lower pore. The coiled‐coil stalk mediates bundling of the four subunits through interactions between predicted *α*‐helices at the base of the channel.

**Table:** 

Nomenclature	TRPA1
HGNC, UniProt	TRPA1, O75762
Chemical activators	Isothiocyanates (covalent) and 1,4‐dihydropyridines (non‐covalent)
Physical activators	Cooling (<17°C) (disputed)
Activators	acrolein (covalent) (pEC_50_ 5.3) [physiological voltage] [22], allicin (covalent) (pEC_50_ 5.1) [physiological voltage] [23], Δ^9^‐tetrahydrocannabinol (non‐covalent) (pEC_50_ 4.9) [‐60mV] [177], nicotine (non‐covalent) (pEC_50_ 4.8) [‐75mV] [400], thymol (non‐covalent) (pEC_50_ 4.7) Concentration range: 6.2×10^−6^M‐2.5×10^−5^M [220], URB597 (non‐covalent) (pEC_50_ 4.6) [287], (‐)‐menthol (Menthol is also active at the mouse TRPA1, but becomes inhibitory at >100μM) (pEC_50_ 4–4.5) [186, 458], cinnamaldehyde (covalent) (pEC_50_ 4.2) [physiological voltage] [14] – Mouse, icilin (non‐covalent) Concentration range: 1×10^−4^M [physiological voltage] [386] – Mouse
Selective activators	chlorobenzylidene malononitrile (covalent) (pEC_50_ 6.7) [41], formalin (covalent. This level of activity is also observed for rat TRPA1) (pEC_50_ 3.4) [253, 266] – Mouse
Channel blockers	AP18 (pIC_50_ 5.5) [328], ruthenium red (pIC_50_ 5.5) [‐80mV] [280] – Mouse, HC030031 (pIC_50_ 5.2) [266]
Functional Characteristics	*γ* = 87–100 pS; conducts mono‐ and di‐valent cations non‐selectively (P_Ca_/PN_a_ = 0.84); outward rectification; activated by elevated intracellular Ca^2+^

### TRPC (canonical) family

Members of the TRPC subfamily (reviewed by [2, 8, 27, 31, 111, 194, 312, 337]) fall into the subgroups outlined below. TRPC2 is a pseudogene in humans. It is generally accepted that all TRPC channels are activated downstream of G_q/11_‐coupled receptors, or receptor tyrosine kinases (reviewed by [333, 415, 455]). A comprehensive listing of G‐protein coupled receptors that activate TRPC channels is given in [2]. Hetero‐oligomeric complexes of TRPC channels and their association with proteins to form signalling complexes are detailed in [8] and [195]. TRPC channels have frequently been proposed to act as store‐operated channels (SOCs) (or compenents of mulimeric complexes that form SOCs), activated by depletion of intracellular calcium stores (reviewed by [8, 61, 321, 334, 359, 475]). However, the weight of the evidence is that they are not directly gated by conventional store‐operated mechanisms, as established for Stim‐gated Orai channels. TRPC channels are not mechanically gated in physiologically relevant ranges of force. All members of the TRPC family are blocked by 2‐APB and SKF96365[139, 140]. Activation of TRPC channels by lipids is discussed by [27].

### TRPC1/C4/C5 subgroup

TRPC4/C5 may be distinguished from other TRP channels by their potentiation by micromolar concentrations of La^3+^. TRPC2 is a pseudogene in humans, but in other mammals appears to be an ion channel localized to microvilli of the vomeronasal organ. It is required for normal sexual behavior in response to pheromones in mice. It may also function in the main olfactory epithelia in mice [236, 304, 305, 472, 473, 474, 487].

### TRPC3/C6/C7 subgroup

All members are activated by diacylglycerol independent of protein kinase C stimulation [140].

**Table:** 

Nomenclature	TRPC1	TRPC2	TRPC3	TRPC4
HGNC, UniProt	TRPC1, P48995	TRPC2, –	TRPC3, Q13507	TRPC4, Q9UBN4
Chemical activators	NO‐mediated cysteine S‐nitrosylation	Diacylglycerol (SAG, OAG, DOG): strongly inhibited by Ca^2+^/CaM once activated by DAG [380]	diacylglycerols	NO‐mediated cysteine S‐nitrosylation, potentiation by extracellular protons
Physical activators	membrane stretch	–	–	–
Endogenous activators	–	Intracellular Ca^2+^	–	–
Activators	–	DOG Concentration range: 1×10^−4^M [‐80mV] [248] – Mouse, SAG Concentration range: 1×10^−4^M [‐80mV] [248] – Mouse	–	La^3+^ (*μ*M range)
Channel blockers	2‐APB [‐70mV] [389], Gd^3+^ Concentration range: 2×10^−5^M [‐70mV] [487], La^3+^ Concentration range: 1×10^−4^M [‐70mV] [389]	2‐APB Concentration range: 5×10^−5^M [‐70mV – 80mV] [248] – Mouse, U73122 (may be indirect) Concentration range: 1×10^−5^M – Mouse	Gd^3+^ (pEC_50_ 7) [‐60mV] [137], BTP2 (pIC_50_ 6.5) [‐80mV] [141], Pyr3 (pIC_50_ 6.2) [197], La^3+^ (pIC_50_ 5.4) [‐60mV] [137], 2‐APB (pIC_50_ 5) [physiological voltage] [234], Ni^2+^, SKF96365	ML204 (pIC_50_ 5.5) [269], La^3+^ (mM range), SKF96365, niflumic acid Concentration range: 3×10^−5^M [‐60mV] [432] – Mouse
Functional Characteristics	It is not yet clear that TRPC1 forms a homomer. It does form heteromers with TRPC4 and TRPC5	*γ* = 42 pS linear single channel conductance in 150 mM symmetrical Na^+^ in vomeronasal sensory neurons. P_Ca_/P_Na_ = 2.7; permeant to Na^+^, Cs^+^, Ca^2+^, but not NMDG [305, 473]	*γ* = 66 pS; conducts mono and di‐valent cations non‐selectively (P_Ca_/P_Na_ = 1.6); monovalent cation current suppressed by extracellular Ca^2+^; dual (inward and outward) rectification	*γ* = 30 –41 pS, conducts mono and di‐valent cations non‐selectively (P_Ca_/P_Na_ = 1.1 – 7.7); dual (inward and outward) rectification

**Table:** 

Nomenclature	TRPC5	TRPC6	TRPC7
HGNC, UniProt	TRPC5, Q9UL62	TRPC6, Q9Y210	TRPC7, Q9HCX4
Chemical activators	NO‐mediated cysteine S‐nitrosylation (disputed), potentiation by extracellular protons	Diacylglycerols	diacylglycerols
Physical activators	Membrane stretch	Membrane stretch	–
Endogenous activators	intracellular Ca^2+^ (at negative potentials) (pEC_50_ 6.2), lysophosphatidylcholine	20‐HETE, arachidonic acid, lysophosphatidylcholine	–
Activators	Gd^3+^ Concentration range: 1×10^−4^M, La^3+^ (*μ*M range), Pb^2+^ Concentration range: 5×10^−6^M, genistein (independent of tyrosine kinase inhibition) [452]	flufenamate, hyp 9 [226], hyperforin [227]	–
Channel blockers	KB‐R7943 (pIC_50_ 5.9) [207], ML204 (pIC_50_∼5) [269], 2‐APB (pIC_50_ 4.7) [‐80mV] [464], La^3+^ Concentration range: 5×10^−3^M [‐60mV] [178] – Mouse	Gd^3+^ (pIC_50_ 5.7) [‐60mV] [164] – Mouse, SKF96365 (pIC_50_ 5.4) [‐60mV] [164] – Mouse, La^3+^ (pIC_50_∼5.2), amiloride (pIC_50_ 3.9) [‐60mV] [164] – Mouse, Cd^2+^ (pIC_50_ 3.6) [‐60mV] [164] – Mouse, 2‐APB, ACAA, GsMTx‐4, Extracellular H^+^, KB‐R7943, ML9	2‐APB, La^3+^ Concentration range: 1×10^−4^M [‐60mV] [303] – Mouse, SKF96365 Concentration range: 2.5×10^−5^M [‐60mV] [303] – Mouse, amiloride
Functional Characteristics	*γ* = 41‐63 pS; conducts mono‐and di‐valent cations non‐selectively (P_Ca_/P_Na_ = 1.8 – 9.5); dual rectification (inward and outward) as a homomer, outwardly rectifying when expressed with TRPC1 or TRPC4	*γ* = 28‐37 pS; conducts mono and divalent cations with a preference for divalents (P_Ca_/P_Na_ = 4.5–5.0); monovalent cation current suppressed by extracellular Ca^2+^ and Mg^2+^, dual rectification (inward and outward), or inward rectification	*γ* = 25–75 pS; conducts mono and divalent cations with a preference for divalents (P_Ca_/ P_Cs_ = 5.9); modest outward rectification (monovalent cation current recorded in the absence of extracellular divalents); monovalent cation current suppressed by extracellular Ca^2+^ and Mg^2+^

### TRPM (melastatin) family

Members of the TRPM subfamily (reviewed by [109, 139, 321, 482]) fall into the five subgroups outlined below.

### TRPM1/M3 subgroup

In darkness, glutamate released by the photoreceptors and ON‐bipolar cells binds to the metabotropic glutamate receptor 6 , leading to activation of Go . This results in the closure of TRPM1. When the photoreceptors are stimulated by light, glutamate release is reduced, and TRPM1 channels are more active, resulting in cell membrane depolarization. Human TRPM1 mutations are associated with congenital stationary night blindness (CSNB), whose patients lack rod function. TRPM1 is also found melanocytes. Isoforms of TRPM1 may present in melanocytes, melanoma, brain, and retina. In melanoma cells, TRPM1 is prevalent in highly dynamic intracellular vesicular structures [165, 298].TRPM3 (reviewed by [301]) exists as multiple splice variants four of which (mTRPM3*α*1, mTRPM3*α*2, hTRPM3a and hTRPM3_1325_) have been characterised and found to differ significantly in their biophysical properties. TRPM3 is expressed in somatosensory neurons and may be important in development of heat hyperalgesia during inflammation. TRPM3 is frequently coexpressed with TRPA1 and TRPV1 in these neurons. TRPM3 is expressed in pancreatic beta cells as well as brain, pituitary gland, eye, kidney, and adipose tissue [300, 408]. TRPM3 may contribute to the detection of noxious heat [428].

### TRPM2

TRPM2 is activated under conditions of oxidative stress (respiratory burst of phagocytic cells) and ischemic conditions. However, the direct activators are ADPR(P) and calcium. As for many ion channels, PIP_2_ must also be present (reviewed by [468]). Numerous splice variants of TRPM2 exist which differ in their activation mechanisms [96]. The C‐terminal domain contains a TRP motif, a coiled‐coil region, and an enzymatic NUDT9 homologous domain. TRPM2 appears not to be activated by NAD, NAAD, or NAADP, but is directly activated by ADPRP (adenosine‐5'‐O‐disphosphoribose phosphate) [417].

### TRPM4/5 subgroup

TRPM4 and TRPM5 have the distinction within all TRP channels of being impermeable to Ca^2+^[455]. A splice variant of TRPM4 (i.e.TRPM4b) and TRPM5 are molecular candidates for endogenous calcium‐activated cation (CAN) channels [130]. TRPM4 is active in the late phase of repolarization of the cardiac ventricular action potential. TRPM4 enhances beta adrenergic‐mediated inotropy. Mutations are associated with conduction defects [170, 263, 381]. TRPM4 has been shown to be an important regulator of Ca^2+^ entry in to mast cells [420] and dendritic cell migration [17]. TRPM5 in taste receptor cells of the tongue appears essential for the transduction of sweet, amino acid and bitter stimuli [235] TRPM5 contributes to the slow afterdepolarization of layer 5 neurons in mouse prefrontal cortex [223].

### TRPM6/7 subgroup

TRPM6 and 7 combine channel and enzymatic activities (‘chanzymes’). These channels have the unusual property of permeation by divalent (Ca^2+^, Mg^2+^, Zn^2+^) and monovalent cations, high single channel conductances, but overall extremely small inward conductance when expressed to the plasma membrane. They are inhibited by internal Mg^2+^ at ∼ 0.6 mM, around the free level of Mg^2+^ in cells. Whether they contribute to Mg^2+^ homeostasis is a contentious issue. When either gene is deleted in mice, the result is embryonic lethality. The C‐terminal kinase region is cleaved under unknown stimuli, and the kinase phosphorylates nuclear histones.

### TRPM8

Is a channel activated by cooling and pharmacological agents evoking a ‘cool’ sensation and participates in the thermosensation of cold temperatures [24, 71, 90] reviewed by [200, 244, 277, 425].

**Table:** 

Nomenclature	TRPM1	TRPM2	TRPM3
HGNC, UniProt	TRPM1, Q7Z4N2	TRPM2, O94759	TRPM3, Q9HCF6
Physical activators	–	Heat ∼ 35°C	heat (Q_10_ = 7.2 between 15 ‐ 25°C; Vriens et al., 2011), hypotonic cell swelling [428]
Endogenous activators	pregnenolone sulphate [216]	intracellular cADPR (pEC_50_ 5) [‐80mV – ‐60mV] [26, 204, 410], intracellular ADP ribose (pEC_50_ 3.9–4.4) [‐80mV] [325], intracellular Ca^2+^ (perhaps via calmodulin), H_2_O_2_ Concentration range: 5×10^−7^M‐5×10^−5^M [physiological voltage] [110, 138, 209, 376, 443], membrane PIP_2_ [416], arachidonic acid Concentration range: 1×10^−5^M‐3×10^−5^M [physiological voltage] [138]	sphingosine (pEC_50_ 4.9) [physiological voltage] [127], epipregnanolone sulphate [259], pregnenolone sulphate [429], sphinganine Concentration range: 2×10^−5^M [physiological voltage] [127]
Activators	–	GEA 3162	nifedipine
Gating inhibitors		–	2‐APB Concentration range: 1×10^−4^M [physiological voltage] [464]
Endogenous channel blockers	Zn^2+^ (pIC_50_ 6)	Zn^2+^ (pIC_50_ 6), extracellular H^+^	Mg^2+^ Concentration range: 9×10^−3^M [‐80mV – 80mV] [299] – Mouse, extracellular Na^+^ (TRPM3*α*2 only)
Channel blockers	–	2‐APB (pIC_50_ 6.1) [‐60mV] [411], ACAA (pIC_50_ 5.8) [physiological voltage] [208], clotrimazole Concentration range: 3×10^−6^M‐3×10^−5^M [‐60mV – ‐15mV] [147], econazole Concentration range: 3×10^−6^M‐3×10^−5^M [‐60mV – ‐15mV] [147], flufenamic acid Concentration range: 5×10^−5^M‐1×10^−3^M [‐60mV – ‐50mV] [146, 411], miconazole Concentration range: 1×10^−5^M [‐60mV] [411]	Gd^3+^ Concentration range: 1×10^−4^M [‐80mV – 80mV] [126, 219], La^3+^ Concentration range: 1×10^−4^M [physiological voltage] [126, 219]
Functional Characteristics	Conducts mono‐ and di‐valent cations non‐selectively, dual rectification (inward and outward)	*γ* = 52‐60 pS at negative potentials, 76 pS at positive potentials; conducts mono‐ and di‐valent cations non‐selectively (P_Ca_/P_Na_ = 0.6‐0.7); non‐rectifying; inactivation at negative potentials; activated by oxidative stress probably via PARP‐1, PARP inhibitors reduce activation by oxidative stress, activation inhibited by suppression of APDR formation by glycohydrolase inhibitors.	TRPM3_1235_: *γ* = 83 pS (Na^+^ current), 65 pS (Ca^2+^ current); conducts mono and di‐valent cations non‐selectively (P_Ca_/P_Na_ = 1.6) TRPM3*α*1: selective for monovalent cations (P_Ca_/P_Cs_ ∼ 0.1); TRPM3*α*2: conducts mono‐ and di‐valent cations non‐selectively (P_Ca_/P_Cs_ = 1–10); Outwardly rectifying (magnitude varies between spice variants)

**Table:** 

Nomenclature	TRPM4	TRPM5	TRPM6
HGNC, UniProt	TRPM4, Q8TD43	TRPM5, Q9NZQ8	TRPM6, Q9BX84
EC number	–	–	2.7.11.1
Other channel blockers	Intracellular nucleotides including ATP, ADP, adenosine 5'‐monophosphate and AMP‐PNP with an IC_50_ range of 1.3‐1.9 *μ*M	–	–
Other chemical activators	–	–	constitutively active, activated by reduction of intracellular Mg^2+^
Physical activators	Membrane depolarization (V_½_ = ‐20 mV to + 60 mV dependent upon conditions) in the presence of elevated [Ca^2+^]_i_, heat (Q_10_ = 8.5 @ +25 mV between 15 and 25°C)	membrane depolarization (V_½_ = 0 to + 120 mV dependent upon conditions), heat (Q_10_ = 10.3 @ ‐75 mV between 15 and 25°C)	–
Endogenous activators	intracellular Ca^2+^ (pEC_50_ 3.9–6.3) [‐100mV – 100mV] [289, 293, 294, 398]	intracellular Ca^2+^ (pEC_50_ 4.5–6.2) [‐80mV – 80mV] [155, 241, 418] – Mouse	extracellular H^+^ (*μ*M range), intracellular Mg^2+^
Activators	BTP2 (pEC_50_ 8.1) [‐80mV] [398], decavanadate (pEC_50_ 5.7) [‐100mV] [293]	–	2‐APB (Potentiation) (pEC_50_ 3.4–3.7) [‐120mV – 100mV] [230]
Gating inhibitors	flufenamic acid (pIC_50_ 5.6) [100mV] [418] – Mouse, clotrimazole Concentration range: 1×10^−6^M‐1×10^−5^M [100mV] [297]	–	–
Endogenous channel blockers	–	–	Mg^2+^ (inward current mediated by monovalent cations is blocked) (pIC_50_ 5.5–6), Ca^2+^ (inward current mediated by monovalent cations is blocked) (pIC_50_ 5.3–5.3)
Channel blockers	9‐phenanthrol (pIC_50_ 4.6–4.8) [122], spermine (pIC_50_ 4.2) [100mV] [295], adenosine (pIC_50_ 3.2)	flufenamic acid (pIC_50_ 4.6), intracellular spermine (pIC_50_ 4.4), Extracellular H^+^ (pIC_50_ 3.2)	ruthenium red (pIC_50_ 7) [voltage dependent ‐120mV]
Functional Characteristics	*γ* = 23 pS (within the range 60 to +60 mV); permeable to monovalent cations; impermeable to Ca^2+^; strong outward rectification; slow activation at positive potentials, rapid deactivation at negative potentials, deactivation blocked by decavanadate	*γ* = 15‐25 pS; conducts monovalent cations selectively (P_Ca_/P_Na_ = 0.05); strong outward rectification; slow activation at positive potentials, rapid inactivation at negative potentials; activated and subsequently desensitized by [Ca^2+^]_I_	*γ*= 40–87 pS; permeable to mono‐ and di‐valent cations with a preference for divalents (Mg^2+^> Ca^2+^; P_Ca_/P_Na_ = 6.9), conductance sequence Zn^2+^> Ba^2+^> Mg^2+^= Ca^2+^ = Mn^2+^> Sr^2+^> Cd^2+^> Ni^2+^; strong outward rectification abolished by removal of extracellular divalents, inhibited by intracellular Mg^2+^ (IC_50_ = 0.5 mM) and ATP
Comments	–	TRPM5 is not blocked by ATP	–

**Table:** 

Nomenclature	TRPM7	TRPM8
HGNC, UniProt	TRPM7, Q96QT4	TRPM8, Q7Z2W7
EC number	2.7.11.1	–
Physical activators	–	depolarization (V_½_ ∼ +50 mV at 15°C), cooling (< 22‐26°C)
Endogenous activators	intracellular ATP, Extracellular H^+^, cyclic AMP (elevated cAMP levels)	–
Activators	2‐APB Concentration range: >1×10^−3^M [279] – Mouse	icilin (pEC_50_ 6.7–6.9) [physiological voltage] [9, 28] – Mouse, (‐)‐menthol (inhibited by intracellular Ca^2+^) (pEC_50_ 4.6) [‐120mV – 160mV] [423]
Selective activators	–	WS‐12 (pEC_50_ 4.9) [physiological voltage] [249, 369] – Rat
Channel blockers	spermine (Reversible, voltage dependent inhibition in RBL2H3 rats) (p*K*_i_ 5.6) [‐110mV – 80mV] [206] – Rat, 2‐APB (Reversible inhibition) (pIC_50_ 3.8) [‐100mV – 100mV] [230] – Mouse, carvacrol (Reversible inhibition) (pIC_50_ 3.5) [‐100mV – 100mV] [310] – Mouse, Mg^2+^ (Reversible inhibition) (pIC_50_ 2.5) [80mV] [279] – Mouse, La^3+^ Concentration range: 2×10^−3^M [‐100mV – 100mV] [356] – Mouse	BCTC (pIC_50_ 6.1) [physiological voltage] [28] – Mouse, 2‐APB (pIC_50_ 4.9–5.1) [100mV – ‐100mV] [157, 284] – Mouse, capsazepine (pIC_50_ 4.7) [physiological voltage] [28] – Mouse
Functional Characteristics	*γ* = 40‐105 pS at negative and positive potentials respectively; conducts mono‐and di‐valent cations with a preference for monovalents (P_Ca_/P_Na_ = 0.34); conductance sequence Ni^2+^> Zn^2+^> Ba^2+^ = Mg^2+^> Ca^2+^ = Mn^2+^> Sr^2+^> Cd^2+^; outward rectification, decreased by removal of extracellular divalent cations; inhibited by intracellular Mg^2+^, Ba^2+^, Sr^2+^, Zn^2+^, Mn^2+^ and Mg.ATP (disputed); activated by and intracellular alkalinization; sensitive to osmotic gradients	*γ* = 40‐83 pS at positive potentials; conducts mono‐ and di‐valent cations non‐selectively (P_Ca_/P_Na_ = 1.0–3.3); pronounced outward rectification; demonstrates densensitization to chemical agonists and adaptation to a cold stimulus in the presence of Ca^2+^; modulated by lysophospholipids and PUFAs
Comments	2‐APB acts as a channel blocker in the μM range.	cannabidiol and Δ^9^‐tetrahydrocannabinol are examples of cannabinoid activators. TRPM8 is insensitive to ruthenium red. icilin requires intracellular Ca^2+^ for full agonist activity.

### TRPML (mucolipin) family

The TRPML family [75, 336, 339, 463, 476] consists of three mammalian members (TRPML1‐3). TRPML channels are probably restricted to intracellular vesicles and mutations in the gene (MCOLN1) encoding TRPML1 (mucolipin‐1) are one cause of the neurodegenerative disorder mucolipidosis type IV (MLIV) in man. TRPML1 is a cation selective ion channel that is important for sorting/transport of endosomes in the late endocytotic pathway and specifically fusion between late endosome‐lysosome hybrid vesicles. TRPML2 and TRPML3 show increased channel activity in low extracellular sodium and are activated by similar small molecules [125]. TRPML3 is important for hair cell maturation, stereocilia maturation and intracellular vesicle transport. A naturally occurring gain of function mutation in TRPML3 (i.e. A419P) results in the varitint waddler (Va) mouse phenotype (reviewed by [292, 339]).

**Table:** 

Nomenclature	TRPML1	TRPML2	TRPML3
HGNC, UniProt	MCOLN1, Q9GZU1	MCOLN2, Q8IZK6	MCOLN3, Q8TDD5
Activators	TRPML1^Va^: Constitutively active, current potentiated by extracellular acidification (equivalent to intralysosomal acidification)	TRPML2^Va^: Constitutively active, current potentiated by extracellular acidification (equivalent to intralysosomal acidification)	TRPML3^Va^: Constitutively active, current inhibited by extracellular acidification (equivalent to intralysosomal acidicification) Wild type TRPML3: Activated by Na^+^‐free extracellular (extracytosolic) solution and membrane depolarization, current inhibited by extracellular acidification (equivalent to intralysosomal acidicification)
Channel blockers			Gd^3+^ (pIC_50_ 4.7) [‐80mV] [281] – Mouse
Functional Characteristics	TRPML1^Va^: *γ* = 40 pS and 76‐86 pS at very negative holding potentials with Fe^2+^ and monovalent cations as charge carriers, respectively; conducts Na^+^≅ K^+^>Cs^+^ and divalent cations (Ba^2+^>Mn2^+^>Fe^2+^>Ca^2+^> Mg^2+^> Ni^2+^>Co^2+^> Cd^2+^>Zn^2+^≫Cu^2+^) protons; monovalent cation flux suppressed by divalent cations (e.g. Ca^2+^, Fe^2+^); inwardly rectifying	TRPML1^Va^: Conducts Na^+^; monovalent cation flux suppressed by divalent cations; inwardly rectifying	TRPML3^Va^: *γ* = 49 pS at very negative holding potentials with monovalent cations as charge carrier; conducts Na^+^> K^+^> Cs^+^ with maintained current in the presence of Na^+^, conducts Ca^2+^ and Mg^2+^, but not Fe^2+^, impermeable to protons; inwardly rectifying Wild type TRPML3: *γ* = 59 pS at negative holding potentials with monovalent cations as charge carrier; conducts Na^+^> K^+^> Cs^+^ and Ca^2+^ (P_Ca_/P_K_≅ 350), slowly inactivates in the continued presence of Na^+^ within the extracellular (extracytosolic) solution; outwardly rectifying

### TRPP (polycystin) family

The TRPP family (reviewed by [87, 89, 118, 153, 451]) or PKD2 family is comprised of PKD2, PKD2L1 and PKD2L2, which have been renamed TRPP1, TRPP2 and TRPP3, respectively [455]. They are clearly distinct from the PKD1 family, whose function is unknown. Although still being sorted out, TRPP family members appear to be 6TM spanning nonselective cation channels.

**Table:** 

Nomenclature	TRPP1	TRPP2	TRPP3
HGNC, UniProt	PKD2, Q13563	PKD2L1, Q9P0L9	PKD2L2, Q9NZM6
Activators	–	Calmidazolium (in primary cilia): 10 μM	–
Channel blockers	–	phenamil (pIC_50_ 6.9), benzamil (pIC_50_ 6), ethylisopropylamiloride (pIC_50_ 5), amiloride (pIC_50_ 3.8), Gd^3+^ Concentration range: 1×10^−4^M [‐50mV] [59], La^3+^ Concentration range: 1×10^−4^M [‐50mV] [59], flufenamate	–
Functional Characteristics	The channel properties of TRPP1 (PKD2) have not been determined	Currents have been measured directly from primary cilia and also when expressed on plasma membranes. Primary cilia appear to contain heteromeric TRPP2 + PKD1‐L1, underlying a gently outwardly rectifying nonselective conductance (P_Ca_/P_Na_ ∼ 6: PKD1‐L1 is a 12 TM protein of unknown topology). Primary cilia heteromeric channels have an inward single channel conductance of 80 pS and an outward single channel conductance of 95 pS. Presumed homomeric TRPP2 channels are gently outwardly rectifying. Single channel conductance is 120 pS inward, 200 pS outward [82].	–

### TRPV (vanilloid) family

Members of the TRPV family (reviewed by [421]) can broadly be divided into the non‐selective cation channels, TRPV1‐4 and the more calcium selective channels TRPV5 and TRPV6.

### TRPV1‐V4 subfamily

TRPV1 is involved in the development of thermal hyperalgesia following inflammation and may contribute to the detection of noxius heat (reviewed by [330, 382, 395]). Numerous splice variants of TRPV1 have been described, some of which modulate the activity of TRPV1, or act in a dominant negative manner when co‐expressed with TRPV1 [366]. The pharmacology of TRPV1 channels is discussed in detail in [132] and [427]. TRPV2 is probably not a thermosensor in man [309], but has recently been implicated in innate immunity [238]. TRPV3 and TRPV4 are both thermosensitive. There are claims that TRPV4 is also mechanosensitive, but this has not been established to be within a physiological range in a native environment [47, 232].

### TRPV5/V6 subfamily

Under physiological conditions, TRPV5 and TRPV6 are calcium selective channels involved in the absorption and reabsorption of calcium across intestinal and kidney tubule epithelia (reviewed by [81, 104, 278, 449]).

**Table:** 

Nomenclature	TRPV1	TRPV2
HGNC, UniProt	TRPV1, Q8NER1	TRPV2, Q9Y5S1
Other chemical activators	NO‐mediated cysteine S‐nitrosylation	–
Physical activators	depolarization (V_½_ ∼ 0 mV at 35°C), noxious heat (> 43°C at pH 7.4)	noxious heat (> 35°C; rodent, not human) [285]
Endogenous activators	extracellular H^+^ (at 37°C) (pEC_50_ 5.4), 12S‐HPETE (pEC_50_ 5.1) [‐60mV] [161] – Rat, 15S‐HPETE (pEC_50_ 5.1) [‐60mV] [161] – Rat, LTB_4_ (pEC_50_ 4.9) [‐60mV] [161] – Rat, 5S‐HETE	–
Activators	resiniferatoxin (pEC_50_ 8.4) [physiological voltage] [374], capsaicin (pEC_50_ 7.5) [‐100mV – 160mV] [423], camphor, diphenylboronic anhydride, phenylacetylrinvanil [12]	2‐APB (pEC_50_ 5) [285, 340] – Rat, Δ^9^‐tetrahydrocannabinol (pEC_50_ 4.8) [340] – Rat, cannabidiol (pEC_50_ 4.5) [340], probenecid (pEC_50_ 4.5) [15] – Rat, 2‐APB (pEC_50_ 3.8–3.9) [physiological voltage] [157, 179] – Mouse, diphenylboronic anhydride Concentration range: 1×10^−4^M [‐80mV] [66, 179] – Mouse
Selective activators	olvanil (pEC_50_ 7.7) [physiological voltage] [374], DkTx (pEC_50_ 6.6) [physiological voltage] [36] – Rat	–
Channel blockers	5'‐iodoresiniferatoxin (pIC_50_ 8.4), 6‐iodo‐nordihydrocapsaicin (pIC_50_ 8), BCTC (pIC_50_ 7.5) [57], capsazepine (pIC_50_ 7.4) [‐60mV] [265], ruthenium red (pIC_50_ 6.7–7)	ruthenium red (pIC_50_ 6.2), TRIM Concentration range: 5×10^−4^M [179] – Mouse
Selective channel blockers	AMG517 (pIC_50_ 9) [35], AMG628 (pIC_50_ 8.4) [435] – Rat, A425619 (pIC_50_ 8.3) [100], A778317 (pIC_50_ 8.3) [30], SB366791 (pIC_50_ 8.2) [134], JYL1421 (pIC_50_ 8) [440] – Rat, JNJ17203212 (pIC_50_ 7.8) [physiological voltage] [393], SB452533 (p*K*_B_ 7.7), SB705498 (pIC_50_ 7.1) [133]	–
Labelled ligands	[^3^H]A778317 (Channel blocker) (p*K*_d_ 8.5) [30], [^125^I]resiniferatoxin (Channel blocker) (pIC_50_ 8.4) [‐50mV] [430] – Rat, [^3^H]resiniferatoxin (Activator)	–
Functional Characteristics	*γ* = 35 pS at – 60 mV; 77 pS at + 60 mV, conducts mono and di‐valent cations with a selectivity for divalents (P_Ca_/P_Na_ = 9.6); voltage‐ and time‐ dependent outward rectification; potentiated by ethanol; activated/potentiated/upregulated by PKC stimulation; extracellular acidification facilitates activation by PKC; desensitisation inhibited by PKA; inhibited by Ca^2+^/ calmodulin; cooling reduces vanilloid‐evoked currents; may be tonically active at body temperature	Conducts mono‐ and di‐valent cations (P_Ca_/P_Na_ = 0.9–2.9); dual (inward and outward) rectification; current increases upon repetitive activation by heat; translocates to cell surface in response to IGF‐1 to induce a constitutively active conductance, translocates to the cell surface in response to membrane stretch

**Table:** 

Nomenclature	TRPV3	TRPV4
HGNC, UniProt	TRPV3, Q8NET8	TRPV4, Q9HBA0
Other chemical activators	NO‐mediated cysteine S‐nitrosylation	Epoxyeicosatrieonic acids and NO‐mediated cysteine S‐nitrosylation
Physical activators	depolarization (V_½_ ∼ +80 mV, reduced to more negative values following heat stimuli), heat (23°C ‐ 39°C, temperature threshold reduces with repeated heat challenge)	Constitutively active, heat (> 24°C ‐ 32°C), mechanical stimuli
Activators	incensole acetate (pEC_50_ 4.8) [273] – Mouse, 2‐APB (pEC_50_ 4.6) [‐80mV – 80mV] [67] – Mouse, diphenylboronic anhydride (pEC_50_ 4.1–4.2) [voltage dependent ‐80mV – 80mV] [66] – Mouse, (‐)‐menthol (pEC_50_ 1.7) [‐80mV – 80mV] [252] – Mouse, camphor Concentration range: 1×10^−3^M‐2×10^−3^M [‐60mV] [271] – Mouse, carvacrol Concentration range: 5×10^−4^M [‐80mV – 80mV] [461] – Mouse, eugenol Concentration range: 3×10^−3^M [‐80mV – 80mV] [461] – Mouse, thymol Concentration range: 5×10^−4^M [‐80mV – 80mV] [461] – Mouse	phorbol 12‐myristate 13‐acetate (pEC_50_ 7.9) [physiological voltage] [459]
Selective activators	6‐tert‐butyl‐m‐cresol (pEC_50_ 3.4) [426] – Mouse	GSK1016790A (pEC_50_ 8.7) [physiological voltage] [409], 4*α*‐PDH (pEC_50_ 7.1) [physiological voltage] [198] – Mouse, RN1747 (pEC_50_ 6.1) [physiological voltage] [422], bisandrographolide (pEC_50_ 6) [‐60mV] [377] – Mouse, 4*α*‐PDD Concentration range: 3×10^−7^M [physiological voltage] [459]
Channel blockers	diphenyltetrahydrofuran (pIC_50_ 5–5.2) [‐80mV – 80mV] [66] – Mouse, ruthenium red Concentration range: 1×10^−6^M [‐60mV] [322] – Mouse	Gd^3+^, La^3+^, ruthenium red Concentration range: 1×10^−6^M [physiological voltage] [172], ruthenium red Concentration range: 2×10^−7^M [physiological voltage] [131] – Rat
Selective channel blockers	–	HC067047 (pIC_50_ 7.3) [‐40mV] [102], RN1734 (pIC_50_ 5.6) [physiological voltage] [422]
Functional Characteristics	*γ* = 197 pS at = +40 to +80 mV, 48 pS at negative potentials; conducts mono‐ and di‐valent cations; outward rectification; potentiated by arachidonic acid	*γ* = ∼ 60 pS at –60 mV, ∼ 90‐100 pS at +60 mV; conducts mono‐ and di‐valent cations with a preference for divalents (P_Ca_/P_Na_ =6–10); dual (inward and outward) rectification; potentiated by intracellular Ca^2+^ via Ca^2+^/ calmodulin; inhibited by elevated intracellular Ca^2+^via an unknown mechanism (IC_50_ = 0.4 μM)

**Table:** 

Nomenclature	TRPV5	TRPV6
HGNC, UniProt	TRPV5, Q9NQA5	TRPV6, Q9H1D0
Other channel blockers	Pb^2+^ = Cu^2+^ = Gd^3+^>Cd^2+^>Zn^2+^>La^3+^>Co^2+^> Fe^2^	–
Activators	constitutively active (with strong buffering of intracellular Ca^2+^)	2‐APB constitutively active (with strong buffering of intracellular Ca^2+^)
Channel blockers	ruthenium red (pIC_50_ 6.9), Mg^2+^	ruthenium red (pIC_50_ 5) [‐80mV] [152] – Mouse, Cd^2+^, La^3+^, Mg^2+^
Functional Characteristics	*γ* = 59–78 pS for monovalent ions at negative potentials, conducts mono‐ and di‐valents with high selectivity for divalents (P_Ca_/P_Na_> 107); voltage‐ and time‐ dependent inward rectification; inhibited by intracellular Ca^2+^ promoting fast inactivation and slow downregulation; feedback inhibition by Ca^2+^ reduced by calcium binding protein 80‐K‐H; inhibited by extracellular and intracellular acidosis; upregulated by 1,25‐dihydrovitamin D3	*γ* = 58–79 pS for monovalent ions at negative potentials, conducts mono‐ and di‐valents with high selectivity for divalents (P_Ca_/P_Na_> 130); voltage‐ and time‐dependent inward rectification; inhibited by intracellular Ca^2+^ promoting fast and slow inactivation; gated by voltage‐dependent channel blockade by intracellular Mg^2+^; slow inactivation due to Ca^2+^‐dependent calmodulin binding; phosphorylation by PKC inhibits Ca^2+^‐calmodulin binding and slow inactivation; upregulated by 1,25‐dihydroxyvitamin D3

### TRPA (ankyrin) family

Agents activating TRPA1 in a covalent manner are thiol reactive electrophiles that bind to cysteine and lysine residues within the cytoplasmic domain of the channel [149, 250]. TRPA1 is activated by a wide range of endogenous and exogenous compounds and only a few representative examples are mentioned in the table: an exhaustive listing can be found in [16]. In addition, TRPA1 is potently activated by intracellular zinc (EC_50_= 8 nM) [10, 156].

### TRPM (melastatin) family

Ca^2+^ activates all splice variants of TRPM2, but other activators listed are effective only at the full length isoform [96]. Inhibition of TRPM2 by clotrimazole, miconazole, econazole, flufenamic acid is largely irreversible. TRPM4 exists as multiple spice variants: data listed are for TRPM4b. The sensitivity of TRPM4b and TRPM5 to activation by [Ca^2+^]_i_ demonstrates a pronounced and time‐dependent reduction following excision of inside‐out membrane patches [418]. The V_½_ for activation of TRPM4 and TRPM5 demonstrates a pronounced negative shift with increasing temperature. Activation of TRPM8 by depolarization is strongly temperature‐dependent via a channel‐closing rate that decreases with decreasing temperature. The V_½_ is shifted in the hyperpolarizing direction both by decreasing temperature and by exogenous agonists, such as (‐)‐menthol[423] whereas antagonists produce depolarizing shifts in V_½_[276]. The V_½_ for the native channel is far more positive than that of heterologously expressed TRPM8 [276]. It should be noted that (‐)‐menthol and structurally related compounds can elicit release of Ca^2+^ from the endoplasmic reticulum independent of activation of TRPM8 [254]. Intracellular pH modulates activation of TRPM8 by cold and icilin, but not (‐)‐menthol[9].

### TRPML (mucolipin) family

Data in the table are for TRPML proteins mutated (i.e TRPML1^Va^, TRPML2^Va^ and TRPML3^Va^) at loci equivalent to TRPML3 A419P to allow plasma membrane expression when expressed in HEK‐293 cells and subsequent characterisation by patch‐clamp recording [94, 123, 191, 281, 462]. Data for wild type TRPML3 are also tabulated [191, 192, 281, 462]. It should be noted that alternative methodologies, particularly in the case of TRPML1, have resulted in channels with differing biophysical characteristics (reviewed by [336]).

### TRPP (polycystin) family

Data in the table are extracted from [79, 89] and [370]. Broadly similar single channel conductance, mono‐ and di‐valent cation selectivity and sensitivity to blockers are observed for TRPP2 co‐expressed with TRPP1 [88]. Ca^2+^, Ba^2+^ and Sr^2+^ permeate TRPP3, but reduce inward currents carried by Na^+^. Mg^2+^ is largely impermeant and exerts a voltage dependent inhibition that increases with hyperpolarization.

### TRPV (vanilloid) family

Activation of TRPV1 by depolarisation is strongly temperature‐dependent via a channel opening rate that increases with increasing temperature. The V_½_ is shifted in the hyperpolarizing direction both by increasing temperature and by exogenous agonists [423]. The sensitivity of TRPV4 to heat, but not 4*α*‐PDD is lost upon patch excision. TRPV4 is activated by anandamide and arachidonic acid following P450 epoxygenase‐dependent metabolism to 5,6‐epoxyeicosatrienoic acid (reviewed by [296]). Activation of TRPV4 by cell swelling, but not heat, or phorbol esters, is mediated via the formation of epoxyeicosatrieonic acids. Phorbol esters bind directly to TRPV4. TRPV5 preferentially conducts Ca^2+^ under physiological conditions, but in the absence of extracellular Ca^2+^, conducts monovalent cations. Single channel conductances listed for TRPV5 and TRPV6 were determined in divalent cation‐free extracellular solution. Ca^2+^‐induced inactivation occurs at hyperpolarized potentials when Ca^2+^ is present extracellularly. Single channel events cannot be resolved (probably due to greatly reduced conductance) in the presence of extracellular divalent cations. Measurements of P_Ca_/P_Na_ for TRPV5 and TRPV6 are dependent upon ionic conditions due to anomalous mole fraction behaviour. Blockade of TRPV5 and TRPV6 by extracellular Mg^2+^ is voltage‐dependent. Intracellular Mg^2+^ also exerts a voltage dependent block that is alleviated by hyperpolarization and contributes to the time‐dependent activation and deactivation of TRPV6 mediated monovalent cation currents. TRPV5 and TRPV6 differ in their kinetics of Ca^2+^‐dependent inactivation and recovery from inactivation. TRPV5 and TRPV6 function as homo‐ and hetero‐tetramers.

### Further reading on Transient Receptor Potential channels

Aghazadeh Tabrizi M et al. (2017) Medicinal Chemistry, Pharmacology, and Clinical Implications of TRPV1 Receptor Antagonists. Med Res Rev 37: 936‐983 [PMID:27976413]

Basso L et al. (2017) Transient Receptor Potential Channels in neuropathic pain. Curr Opin Pharmacol 32: 9‐15 [PMID:27835802]

Ciardo MG et al. (2017) Lipids as central modulators of sensory TRP channels. Biochim Biophys Acta 1859: 1615‐1628 [PMID:28432033]

Clapham DE et al. (2003) International Union of Pharmacology. XLIII. Compendium of voltage‐gated ion channels: transient receptor potential channels. Pharmacol Rev 55: 591‐6 [PMID:14657417]

Diaz‐Franulic I et al. (2016) Allosterism and Structure in Thermally Activated Transient Receptor Potential Channels. Annu Rev Biophys 45: 371‐98 [PMID:27297398]

Grace MS et al. (2017) Modulation of the TRPV4 ion channel as a therapeutic target for disease. Pharmacol Ther [PMID:28202366]

Grayson TH et al. (2017) Transient receptor potential canonical type 3 channels: Interactions, role and relevance ‐ A vascular focus. Pharmacol Ther 174: 79‐96 [PMID:28223224]

Kashio M et al. (2017) The TRPM2 channel: a thermo‐sensitive metabolic sensor. Channels (Austin) 0 [PMID:28633002]

Wu LJ et al. (2010) International Union of Basic and Clinical Pharmacology. LXXVI. Current progress in the mammalian TRP ion channel family. Pharmacol Rev 62: 381‐404 [PMID:20716668]

Zierler S et al. (2017) TRPM channels as potential therapeutic targets against pro‐inflammatory diseases. Cell Calcium [PMID:28549569]

## Voltage‐gated calcium channels

### Overview

Calcium (Ca^2+^) channels are voltage‐gated ion channels present in the membrane of most excitable cells. The nomenclature for Ca^2+^channels was proposed by [101] and **approved by the** **NC‐IUPHAR** **Subcommittee on Ca^2+^ channels [** **54** **]**. Ca^2+^ channels form hetero‐oligomeric complexes. The *α*1 subunit is pore‐forming and provides the binding site(s) for practically all agonists and antagonists. The 10 cloned *α*1‐subunits can be grouped into three families: (1) the high‐voltage activated dihydropyridine‐sensitive (L‐type, Ca_V_1.x) channels; (2) the high‐voltage activated dihydropyridine‐insensitive (Ca_V_2.x) channels and (3) the low‐voltage‐activated (T‐type, Ca_V_3.x) channels. Each *α*1 subunit has four homologous repeats (I–IV), each repeat having six transmembrane domains and a pore‐forming region between transmembrane domains S5 and S6. Gating is thought to be associated with the membrane‐spanning S4 segment, which contains highly conserved positive charges. Many of the *α*1‐subunit genes give rise to alternatively spliced products. At least for high‐voltage activated channels, it is likely that native channels comprise co‐assemblies of *α*1, *β* and *α*2–*δ* subunits. The *γ* subunits have not been proven to associate with channels other than the *α*1s skeletal muscle Cav1.1 channel. The *α*2–*δ*1 and *α*2–*δ*2 subunits bind gabapentin and pregabalin.

**Table:** 

Nomenclature	Ca_v_1.1	Ca_v_1.2	Ca_v_1.3	Ca_v_1.4
HGNC, UniProt	CACNA1S, Q13698	CACNA1C, Q13936	CACNA1D, Q01668	CACNA1F, O60840
Activators	FPL64176 (pEC_50_∼7.8), (‐)‐(S)‐BayK8644 (pEC_50_∼7.8)	(‐)‐(S)‐BayK8644 (pEC_50_∼7.8), FPL64176 Concentration range: 1×10^−6^M‐5×10^−6^M [243] – Rat	FPL64176 (pEC_50_∼7.8), (‐)‐(S)‐BayK8644 (pEC_50_∼7.8)	(‐)‐(S)‐BayK8644 (pEC_50_∼7.8)
Gating inhibitors	nifedipine (pIC_50_ 6.3) Concentration range: 1×10^−7^M‐1×10^−4^M [voltage dependent ‐90mV] [215] – Rat, nimodipine (pIC_50_∼6) [‐70mV], nitrendipine (pIC_50_ 6) [‐80mV] [25] – Rat	nifedipine (pIC_50_ 7.7) [‐80mV] [329] – Rat, nimodipine (pIC_50_ 6.8) [‐80mV] [465] – Rat, nitrendipine (pIC_50_ 6) [‐80mV] [465] – Rat	nitrendipine (pIC_50_ 8.4) [373], nifedipine (pIC_50_ 7.7) [373], nimodipine (pIC_50_ 5.7–6.6) [‐80mV – ‐40mV] [357, 465] – Rat	nifedipine (pIC_50_ 6) [‐100mV] [267], nimodipine (pIC_50_∼6) [‐70mV], nitrendipine (pIC_50_∼6) [‐70mV]
Selective gating inhibitors	–	–	–	–
Channel blockers	diltiazem, verapamil	diltiazem, verapamil	verapamil	diltiazem (pIC_50_ 4) [‐80mV] [21] – Mouse, verapamil Concentration range: 1×10^−4^M [‐80mV] [21] – Mouse
Sub/family‐selective channel blockers	calciseptine	calciseptine	–	–
Functional Characteristics	L‐type calcium current: High voltage‐activated, slow voltage dependent inactivation	L‐type calcium current: High voltage‐activated, slow voltage‐dependent inactivation, rapid calcium‐dependent inactivation	L‐type calcium current: Voltage‐activated, slow voltage‐dependent inactivation, more rapid calcium‐dependent inactivation	L‐type calcium current: Moderate voltage‐activated, slow voltage‐dependent inactivation
Comments	–	–	Ca_v_1.3 activates more negative potentials than Ca_v_1.2 and is incompletely inhibited by dihydropyridine antagonists.	Ca_v_1.4 is less sensitive to dihydropyridine antagonists than other Cav1 channels

**Table:** 

Nomenclature	Ca_v_2.1	Ca_v_2.2	Ca_v_2.3
HGNC, UniProt	CACNA1A, O00555	CACNA1B, Q00975	CACNA1E, Q15878
Selective gating inhibitors	*ω*‐agatoxin IVA (P current component: Kd = ∼ 2nM, Q component Kd= >100nM) (pIC_50_ 7–8.7) [‐100mV – ‐90mV] [38, 270] – Rat, *ω*‐agatoxin IVB (p*K*_d_ 8.5) [‐80mV] [4] – Rat	–	SNX482 (pIC_50_ 7.5–8) [physiological voltage] [286]
Channel blockers	–	–	Ni^2+^ (pIC_50_ 4.6) [‐90mV] [448]
Sub/family‐selective channel blockers	*ω*‐conotoxin MVIIC (pIC_50_ 8.2–9.2) Concentration range: 2×10^−6^M‐5×10^−6^M [physiological voltage] [229] – Rat	*ω*‐conotoxin GVIA (pIC_50_ 10.4) [‐80mV] [229] – Rat, *ω*‐conotoxin MVIIC (pIC_50_ 6.1–8.5) [‐80mV] [148, 229, 264] – Rat	–
Functional Characteristics	P/Q‐type calcium current: Moderate voltage‐activated, moderate voltage‐dependent inactivation	N‐type calcium current: High voltage‐activated, moderate voltage‐dependent inactivation	R‐type calcium current: Moderate voltage‐activated, fast voltage‐dependent inactivation

**Table:** 

Nomenclature	Ca_v_3.1	Ca_v_3.2	Ca_v_3.3
HGNC, UniProt	CACNA1G, O43497	CACNA1H, O95180	CACNA1I, Q9P0X4
Gating inhibitors	kurtoxin (pIC_50_ 7.3–7.8) [‐90mV] [63, 371] – Rat	kurtoxin (pIC_50_ 7.3–7.6) [‐90mV] [63, 371] – Rat	–
Channel blockers	mibefradil (pIC_50_ 6–6.6) [‐110mV – ‐100mV] [261], Ni^2+^ (pIC_50_ 3.6–3.8) [voltage dependent ‐90mV] [218] – Rat	mibefradil (pIC_50_ 5.9–7.2) [‐110mV – ‐80mV] [261], Ni^2+^ (pIC_50_ 4.9–5.2) [voltage dependent ‐90mV] [218]	mibefradil (pIC_50_ 5.8) [‐110mV] [261], Ni^2+^ (pIC_50_ 3.7–4.1) [voltage dependent ‐90mV] [218] – Rat
Functional Characteristics	T‐type calcium current: Low voltage‐activated, fast voltage‐dependent inactivation	T‐type calcium current: Low voltage‐activated, fast voltage‐dependent inactivation	T‐type calcium current: Low voltage‐activated, moderate voltage‐dependent inactivation

### Comments

In many cell types, P and Q current components cannot be adequately separated and many researchers in the field have adopted the terminology ‘P/Q‐type’ current when referring to either component. Both of these physiologically defined current types are conducted by alternative forms of Cav2.1. Ziconotide (a synthetic peptide equivalent to *ω*‐conotoxin MVIIA) has been approved for the treatment of chronic pain [447].

### Further reading on Voltage‐gated calcium channels

Catterall WA et al. (2015) Structural Basis for Pharmacology of Voltage‐Gated Sodium and Calcium Channels. Mol Pharmacol 88: 141‐50 [PMID:25848093]

Catterall WA et al. (2005) International Union of Pharmacology. XLVIII. Nomenclature and structure‐function relationships of voltage‐gated calcium channels. Pharmacol Rev 57: 411‐25 [PMID:16382099]

Catterall WA et al. (2015) Deciphering voltage‐gated Na(+) and Ca(2+) channels by studying prokaryotic ancestors. Trends Biochem Sci 40: 526‐34 [PMID:26254514]

Dolphin AC. (2016) Voltage‐gated calcium channels and their auxiliary subunits: physiology and pathophysiology and pharmacology. J Physiol 594: 5369‐90 [PMID:27273705]

Huang J et al. (2017) Regulation of voltage gated calcium channels by GPCRs and post‐translational modification. Curr Opin Pharmacol 32: 1‐8 [PMID:27768908]

Ortner NJ et al. (2016) L‐type calcium channels as drug targets in CNS disorders. Channels (Austin) 10: 7‐13 [PMID:26039257]

Rougier JS et al. (2016) Cardiac voltage‐gated calcium channel macromolecular complexes. Biochim Biophys Acta 1863: 1806‐12 [PMID:26707467]

Zamponi GW. (2016) Targeting voltage‐gated calcium channels in neurological and psychiatric diseases. Nat Rev Drug Discov 15: 19‐34 [PMID:26542451]

## Voltage‐gated proton channel

### Overview

The voltage‐gated proton channel (provisionally denoted H_v_1) is a putative 4TM proton‐selective channel gated by membrane depolarization and which is sensitive to the transmembrane pH gradient [49, 84, 85, 346, 362]. The structure of H_v_1 is homologous to the voltage sensing domain (VSD) of the superfamily of voltage‐gated ion channels (i.e. segments S1 to S4) and contains no discernable pore region [346, 362]. Proton flux through H_v_1 is instead most likely mediated by a water wire completed in a crevice of the protein when the voltage‐sensing S4 helix moves in response to a change in transmembrane potential [345, 453]. H_v_1 expresses largely as a dimer mediated by intracellular C‐terminal coiled‐coil interactions [231] but individual promoters nonetheless support gated H^+^ flux via separate conduction pathways [203, 221, 327, 412]. Within dimeric structures, the two protomers do not function independently, but display co‐operative interactions during gating resulting in increased voltage sensitivity, but slower activation, of the dimeric,versus monomeric, complexes [121, 413].

**Table:** 

Nomenclature	H_v_1
HGNC, UniProt	HVCN1, Q96D96
Channel blockers	Zn^2+^ (pIC_50_∼5.7–6.3), Cd^2+^ (pIC_50_∼5)
Functional Characteristics	Activated by membrane depolarization mediating macroscopic currents with time‐, voltage‐ and pH‐dependence; outwardly rectifying; voltage dependent kinetics with relatively slow current activation sensitive to extracellular pH and temperature, relatively fast deactivation; voltage threshold for current activation determined by pH gradient (ΔpH = pH_o_ ‐pH_i_) across the membrane

### Comments

The voltage threshold (V_thr_) for activation of Hv1 is not fixed but is set by the pH gradient across the membrane such that V_thr_ is positive to the Nernst potential for H^+^, which ensures that only outwardly directed flux of H^+^ occurs under physiological conditions [49, 84, 85]. Phosphorylation of H_v_1 within the N‐terminal domain by PKC enhances the gating of the channel [274]. Tabulated IC_50_ values for Zn^2^+ and Cd^2+^ are for heterologously expressed human and mouse H_v_1 [346, 362]. Zn^2+^ is not a conventional pore blocker, but is coordinated by two, or more, external protonation sites involving histamine residues [346]. Zn^2+^ binding may occur at the dimer interface between pairs of histamine residues from both monomers where it may interfere with channel opening [275]. Mouse knockout studies demonstrate that H_v_1 participates in charge compensation in granulocytes during the respiratory burst of NADPH oxidase‐dependent reactive oxygen species production that assists in the clearance of bacterial pathogens [347]. Additional physiological functions of H_v_1 are reviewed by [49].

### Further reading on Voltage‐gated proton channel

Castillo K et al. (2015) Voltage‐gated proton (H(v)1) channels, a singular voltage sensing domain. FEBS Lett 589: 3471‐8 [PMID:26296320]

DeCoursey TE. (2015) The Voltage‐Gated Proton Channel: A Riddle, Wrapped in a Mystery, inside an Enigma. Biochemistry 54: 3250‐68 [PMID:25964989]

DeCoursey TE. (2013) Voltage‐gated proton channels: molecular biology, physiology, and pathophysiology of the H(V) family. Physiol Rev 93: 599‐652 [PMID:23589829]

Fernandez A et al. (2016) Pharmacological Modulation of Proton Channel Hv1 in Cancer Therapy: Future Perspectives. Mol Pharmacol 90: 385‐402 [PMID:27260771]

Okamura Y et al. (2015) Gating mechanisms of voltage‐gated proton channels. Annu Rev Biochem 84: 685‐709 [PMID:26034892]

## Voltage‐gated sodium channels

### Overview

Sodium channels are voltage‐gated sodium‐selective ion channels present in the membrane of most excitable cells. Sodium channels comprise of one pore‐forming *α* subunit, which may be associated with either one or two *β* subunits [169]. *α*‐Subunits consist of four homologous domains (I–IV), each containing six transmembrane segments (S1–S6) and a pore‐forming loop. The positively charged fourth transmembrane segment (S4) acts as a voltage sensor and is involved in channel gating. The crystal structure of the bacterial NavAb channel has revealed a number of novel structural features compared to earlier potassium channel structures including a short selectivity filter with ion selectivity determined by interactions with glutamate side chains [316]. Interestingly, the pore region is penetrated by fatty acyl chains that extend into the central cavity which may allow the entry of small, hydrophobic pore‐blocking drugs [316]. Auxiliary *β*1, *β*2, *β*3 and *β*4 subunits consist of a large extracellular N‐terminal domain, a single transmembrane segment and a shorter cytoplasmic domain.

**The nomenclature for sodium channels was proposed by Goldin et al., (2000) [** **119** **] and approved by the** **NC‐IUPHAR** **Subcommittee on sodium channels (Catterall et al., 2005, [** **52** **])**.

**Table:** 

Nomenclature	Na_v_1.1	Na_v_1.2	Na_v_1.3	Na_v_1.4
HGNC, UniProt	SCN1A, P35498	SCN2A, Q99250	SCN3A, Q9NY46	SCN4A, P35499
Sub/family‐selective activators	batrachotoxin, veratridine	batrachotoxin (p*K*_d_ 9.1) [physiological voltage] [237] – Rat, veratridine (p*K*_d_ 5.2) [physiological voltage] [53] – Rat	batrachotoxin, veratridine	batrachotoxin Concentration range: 5×10^−6^M [‐100mV] [438] – Rat, veratridine Concentration range: 2×10^−4^M [‐100mV] [438] – Rat
Channel blockers	tetrodotoxin (p*K*_d_ 8) [‐100mV] [378] – Rat	–	–	–
Sub/family‐selective channel blockers	Hm1a [306] – Rat, saxitoxin	saxitoxin (pIC_50_ 8.8) [‐120mV] [40] – Rat, tetrodotoxin (pIC_50_ 8) [‐120mV] [40] – Rat, lacosamide (pIC_50_ 4.5) [‐80mV] [1] – Rat	tetrodotoxin (pIC_50_ 8.4) [60], saxitoxin	saxitoxin (pIC_50_ 8.4) [‐100mV] [324] – Rat, tetrodotoxin (pIC_50_ 7.6) [‐120mV] [56], *μ*‐conotoxin GIIIA (pIC_50_ 5.9) [‐100mV] [56]
Functional Characteristics	Activation V_0.5_ = ‐20 mV. Fast inactivation (*τ* = 0.7 ms for peak sodium current).	Activation V_0.5_ = ‐24 mV. Fast inactivation (*τ* = 0.8 ms for peak sodium current).	Activation V_0.5_ = ‐24 mV. Fast inactivation (0.8 ms)	Activation V_0.5_ = ‐30 mV. Fast inactivation (0.6 ms)

**Table:** 

Nomenclature	Na_v_1.5	Na_v_1.6	Na_v_1.7	Na_v_1.8	Na_v_1.9
HGNC, UniProt	SCN5A, Q14524	SCN8A, Q9UQD0	SCN9A, Q15858	SCN10A, Q9Y5Y9	SCN11A, Q9UI33
Sub/family‐selective activators	batrachotoxin (p*K*_d_ 7.6) [physiological voltage] [368] – Rat, veratridine (pEC_50_ 6.3) [‐30mV] [433] – Rat	batrachotoxin, veratridine	batrachotoxin, veratridine	–	–
Sub/family‐selective channel blockers	tetrodotoxin (p*K*_d_ 5.8) [‐80mV] [74, 477] – Rat	tetrodotoxin (pIC_50_ 9) [‐130mV] [91] – Rat, saxitoxin	tetrodotoxin (pIC_50_ 7.6) [‐100mV] [199], saxitoxin (pIC_50_ 6.2) [431]	tetrodotoxin (pIC_50_ 4.2) [‐60mV] [5] – Rat	tetrodotoxin (pIC_50_ 4.4) [‐120mV] [76] – Rat
Selective channel blockers	–	–	–	PF‐01247324 (pIC_50_ 6.7) [voltage dependent] [317]	–
Functional Characteristics	Activation V_0.5_ = ‐26 mV. Fast inactivation (*τ* = 1 ms for peak sodium current).	Activation V_0.5_ = ‐29 mV. Fast inactivation (1 ms)	Activation V_0.5_ = ‐27 mV. Fast inactivation (0.5 ms)	Activation V_0.5_ = ‐16 mV. Inactivation (6 ms)	Activation V_0.5_ = ‐32 mV. Slow inactivation (16 ms)

### Comments

Sodium channels are also blocked by local anaesthetic agents, antiarrythmic drugs and antiepileptic drugs. In general, these drugs are not highly selective among channel subtypes. There are two clear functional fingerprints for distinguishing different subtypes. These are sensitivity to tetrodotoxin(Na_V_1.5, Na_V_1.8 and Na_V_1.9 are much less sensitive to block) and rate of fast inactivation (Na_V_1.8 and particularly Na_V_1.9 inactivate more slowly). All sodium channels also have a slow inactivation process that is engaged during long depolarizations (>100 msec) or repetitive trains of stimuli. All sodium channel subtypes are blocked by intracellular QX‐314.

### Further reading on Voltage‐gated sodium channels

Catterall WA et al. (2005) International Union of Pharmacology. XLVII. Nomenclature and structure‐function relationships of voltage‐gated sodium channels. Pharmacol Rev 57: 397‐409 [PMID:16382098]

Catterall WA et al. (2017) The chemical basis for electrical signaling. Nat Chem Biol 13: 455‐463 [PMID:28406893]

Deuis JR et al. (2017) The pharmacology of voltage‐gated sodium channel activators. Neuropharmacology [PMID:28416444]

Kanellopoulos AH et al. (2016) Voltage‐gated sodium channels and pain‐related disorders. Clin Sci (Lond) 130: 2257‐2265 [PMID:27815510]

Terragni B et al. (2017) Post‐translational dysfunctions in channelopathies of the nervous system. Neuropharmacology [PMID:28571716]
